# Optimal Duration of Dual Antiplatelet Therapy After Percutaneous Coronary Intervention of the Left Main Coronary Artery: A Contemporary Narrative Review

**DOI:** 10.3390/medicina62081487

**Published:** 2026-08-01

**Authors:** Daniel Miron Brie, Cristian Mornoș, Roxana Popescu, Alina Diduța Brie

**Affiliations:** 1 Cardiovascular Disease Institute Timisoara, Gheorghe Adam Street, No. 13A, 300310 Timisoara, Romania; mornos.cristian@umft.ro; 2 Research Center of the Institute of Cardiovascular Diseases, Gheorghe Adam Street, No. 13A, 300310 Timișoara, Romania; 3 Department of Cardiology, “Victor Babeș” University of Medicine and Pharmacy Timisoara, Eftimie Murgu Square, No. 2, 300041 Timisoara, Romania; 4 Department of Cell and Molecular Biology, “Victor Babeș” University of Medicine and Pharmacy, Tudor Vladimirescu Street, No. 14, 300174 Timisoara, Romania; popescu.roxana@umft.ro (R.P.); alina.brie@umft.ro (A.D.B.); 5 ANAPATMOL Research Center, “Victor Babeș” University of Medicine and Pharmacy, Tudor Vladimirescu Street, No. 14, 300174 Timisoara, Romania; 6 “Louis Țurcanu” Emergency Children Hospital, Doctor Iosif Nemoianu Street, No. 2, 300011 Timisoara, Romania

**Keywords:** left main coronary artery, dual antiplatelet therapy, percutaneous coronary intervention, bifurcation lesion, bleeding risk, ischemic risk, drug-eluting stent, P2Y_12_ inhibitor, PARTHENOPE, NEO-MINDSET, intravascular imaging

## Abstract

*Background*: The optimal duration of dual antiplatelet therapy (DAPT) after percutaneous coronary intervention (PCI) of the left main coronary artery remains uncertain because this lesion involves a large myocardial territory and requires a careful balance between ischemic protection and bleeding risk. This review aimed to provide an updated, left-main-focused synthesis of the evidence on DAPT duration after PCI and to clarify how treatment should be individualized according to clinical presentation, lesion complexity, procedural strategy, intravascular imaging, and validated ischemic and bleeding risk scores. *Methods*: This narrative review with a structured literature search examined studies published from January 2010 through May 2026 in PubMed/MEDLINE, EMBASE, the Cochrane Central Register of Controlled Trials, and Google Scholar. From 248 identified records, 62 studies met the eligibility criteria and were included in the qualitative synthesis, comprising 38 randomized controlled trials and 24 observational studies or pooled analyses. The review prioritized direct left-main-specific evidence while also incorporating broader PCI studies with left-main subgroups and indirect contextual evidence relevant to antiplatelet decision-making. Independent screening, duplicate data extraction, and qualitative risk-of-bias assessment were performed, but the review was not prospectively registered, and no meta-analysis was conducted. *Results*: The available evidence supports an individualized rather than fixed DAPT strategy after left main PCI. In stable patients with anatomically simple left main lesions and acceptable bleeding risk, 6–12 months of DAPT appears generally sufficient, whereas patients with acute coronary syndromes, two-stent distal bifurcation strategies, high thrombotic burden, or other high-ischemic-risk features may derive greater benefit from extending therapy beyond 12 months when bleeding risk is low. Contemporary guideline recommendations are broadly aligned with this risk-adapted approach, and recent trials further refine decision-making: PARTHENOPE provided randomized support for risk-score-guided personalization of DAPT duration, whereas NEO-MINDSET cautioned against immediate aspirin withdrawal after PCI in acute coronary syndromes. Intravascular imaging, especially IVUS and OCT, improves procedural optimization and may help contextualize post-PCI thrombotic risk, although current data do not validate imaging findings alone as a stand-alone criterion for abbreviated DAPT. *Conclusions*: DAPT duration after left main PCI should be individualized by integrating clinical presentation, lesion and procedural complexity, intravascular imaging, and validated ischemic and bleeding risk tools. A personalized, risk-adapted strategy currently offers the most appropriate framework for balancing ischemic benefit against bleeding harm in this high-risk population.

## 1. Introduction

Antithrombotic management after percutaneous coronary intervention (PCI) of the left main (LM) coronary artery sits at the crossroads of two competing harms: catastrophic ischemic events arising from a single, large-territory vessel and life-threatening bleeding driven by prolonged platelet inhibition. Although complex PCI—LM and bifurcation lesions in particular—now accounts for a sizeable share of contemporary interventions, no single duration of dual antiplatelet therapy (DAPT) has emerged as universally appropriate. Decisions are shaped by clinical presentation, baseline ischemic and bleeding risk, and the specific stenting strategy used [1]. Recent registries, randomized trials, and meta-analyses suggest that an individualized DAPT duration—rather than a fixed regimen—offers the most favorable net clinical benefit, particularly in anatomically high-risk lesions [2,3,4]. In this review, DAPT duration is operationally classified as short (≤6 months), standard (>6 to 12 months), and prolonged (>12 months), in line with contemporary guideline-based and trial-based usage.

Despite a substantial body of literature, the optimal duration of DAPT after LM stenting remains contested, with conflicting signals across efficacy and safety endpoints. Three concepts underpin contemporary decision-making: stratifying patients by clinical and procedural risk, accounting for stenting complexity (notably bifurcation lesions treated with one- or two-stent techniques), and applying validated risk scores, such as DAPT and PRECISE-DAPT, to guide duration [5]. The 2025 PARTHENOPE [6] randomized trial—the first to directly compare risk-score-guided personalization with a fixed 12-month strategy—has provided pivotal new evidence supporting this paradigm and is integrated throughout this review.

Any contemporary discussion of antiplatelet therapy after LM PCI must be anchored in the two landmark revascularization trials that defined the modern indications for percutaneous treatment of this anatomy. In the EXCEL [7] trial, Stone and colleagues randomized 1905 patients with LM disease of low-to-intermediate anatomic complexity (SYNTAX score ≤ 32) to PCI with a fluoropolymer-based everolimus-eluting stent or to coronary artery bypass grafting (CABG); at five years, the primary composite of death, stroke or myocardial infarction occurred in 22.0% of PCI-treated patients and in 19.2% of those who underwent CABG, supporting non-inferiority of PCI although all-cause mortality was numerically higher with PCI (13.0% vs. 9.9%). In the parallel NOBLE [8] trial, Holm and co-investigators initially randomized 1201 patients to PCI with predominantly biolimus-eluting stents or to CABG and at five years observed a MACCE rate of 28% versus 19% (hazard ratio 1.58; 95% CI 1.24–2.01; *p* = 0.0002). The final 10-year mortality results of NOBLE [9], presented at TCT 2025 and published in the Lancet in 2026 by Christiansen and colleagues, reported all-cause mortality of 23% with PCI versus 25% with CABG (HR 0.93; 95% CI 0.74–1.18; *p* = 0.56), with no significant difference in cardiovascular mortality and a signal of lower mortality with PCI specifically in patients with acute coronary syndromes (HR 0.57; 95% CI 0.32–0.99; *p* = 0.047). Together with the 10-year SYNTAX Extended Survival and PRECOMBAT data, these results indicate that PCI with contemporary drug-eluting stents is as safe as CABG over a decade of follow-up for appropriately selected LM patients—reinforcing the central importance of optimal antithrombotic therapy in this high-stakes subset, since any ischemic event after LM PCI carries a substantially worse prognosis than after PCI of non-LM territories.

This review includes several elements that, to our knowledge, have not been integrated into prior LM-focused DAPT overviews, such as those by Brener et al. [10], Carciotto et al. [11], or Hartikainen et al. [12]. Specifically, it is the first LM-centric synthesis to incorporate the PARTHENOPE [6] trial and to develop a risk-score-anchored, quadrant-based algorithm to personalize DAPT duration after LM PCI. It also uniquely integrates intravascular imaging data from OCTOBER [13] and ILUMIEN IV [14], together with the final 10-year mortality results from NOBLE [9], thereby linking contemporary device optimization and long-term revascularization outcomes to practical DAPT decisions in LM disease. Collectively, these features position the present work as a contemporary, algorithm-driven update rather than a simple extension of earlier reviews. This review aims to provide an updated LM-focused synthesis of DAPT duration after PCI by integrating direct LM studies, relevant LM subgroup analyses, and selected contextual evidence from broader contemporary PCI trials. Its principal contribution is not to establish definitive duration thresholds, but to clarify the directness, strengths, and limitations of the evidence currently informing individualized DAPT decisions in LM disease.

### 1.1. Objectives

The present review pursues five interlocking objectives: (i) to appraise current evidence on clinical outcomes associated with varying DAPT durations after LM stenting; (ii) to benchmark contemporary guideline recommendations on DAPT duration and antiplatelet agent selection in this setting; (iii) to identify demographic and clinical factors that influence DAPT duration decisions; (iv) to compare the efficacy and safety profiles of short-term, standard and extended regimens, including risk-score-personalized durations; and (v) to analyze how procedural strategies—particularly bifurcation stenting techniques and intravascular imaging—interact with DAPT duration to shape prognosis. This review is best characterized as a narrative review with a structured literature search, rather than a fully systematic review. It employs predefined eligibility criteria, multiple databases, and a PRISMA-style flow diagram to ensure comprehensive coverage, but it was not prospectively registered and does not adhere to all PRISMA requirements. Formal risk-of-bias tools (ROBINS-I and RoB 2) were applied qualitatively, and no quantitative meta-analysis was conducted. Accordingly, all conclusions should be interpreted within the framework of an evidence-based narrative synthesis, not a formal systematic review.

### 1.2. Research Question

What is the optimal duration of dual antiplatelet therapy for adults undergoing percutaneous coronary intervention for left main coronary artery disease, and how should it be personalized based on patient, lesion, and procedural characteristics?

## 2. Materials and Methods

This contemporary narrative review examined the optimal duration of dual antiplatelet therapy after percutaneous coronary intervention of the left main coronary artery. From an initial pool of 248 records, 62 high-quality studies (38 randomized controlled trials and 24 observational studies or pooled analyses) met our eligibility criteria and form the core of the synthesis.

### 2.1. Literature Search and Study Selection

PubMed/MEDLINE (U.S. National Library of Medicine, Bethesda, MD, USA; https://pubmed.ncbi.nlm.nih.gov, accessed 31 May 2026), EMBASE (Elsevier, Amsterdam, The Netherlands; https://www.embase.com, accessed 31 May 2026), the Cochrane Central Register of Controlled Trials (CENTRAL; Cochrane, London, UK; https://www.cochranelibrary.com, accessed 31 May 2026), and Google Scholar (Google LLC, Mountain View, CA, USA; https://scholar.google.com, accessed 31 May 2026) were searched from 1 January 2010, through 31 May 2026; 31 May 2026, was considered the final search cut-off date for all databases.

The search strategy combined four conceptual blocks—anatomy, intervention, antiplatelet therapy, and outcomes—using the following terms and their variants: “left main” OR “LMCA” OR “unprotected left main”; “PCI” OR “percutaneous coronary intervention” OR “stenting”; “DAPT” OR “dual antiplatelet therapy” OR “P2Y12 inhibitor” OR “antiplatelet”; “duration” OR “short-term” OR “long-term” OR “extended” OR “personalized”; and “outcomes” OR “mortality” OR “MACE” OR “bleeding” OR “stent thrombosis”. Reference lists of included articles, recent ESC and ACC/AHA guidelines, and major late-breaking trial presentations from ESC 2024–2025, TCT 2024–2025, and ACC 2024–2025 were hand-searched to identify additional relevant studies, including late-breaking trials not yet indexed at the time of the original database queries.

Two reviewers independently screened titles and abstracts against predefined eligibility criteria, with disagreements resolved by discussion and, when necessary, adjudication by a third reviewer. Full-text assessment of potentially eligible reports was likewise performed independently by two reviewers, and reasons for exclusion were documented systematically and summarized in a PRISMA-style flow diagram. Data extraction from all included studies was conducted in duplicate using a standardized template that captured study design, population characteristics, anatomical and procedural features, DAPT duration categories, clinical endpoints, and follow-up duration; discrepancies were resolved by consensus and, where needed, by consulting the original article.

Unpublished data and grey literature were not systematically sought beyond late-breaking trial presentations at major cardiology congresses (ESC, ACC, TCT), which were subsequently traced to full-text peer-reviewed publications when available. Abstract-only reports, conference proceedings without full-text manuscripts, and non–peer-reviewed sources were excluded unless a corresponding full publication could be identified during the search update. A synoptic overview of the randomized evidence is provided in Supplementary Table S1, which classifies each trial according to LM sample size or subgroup availability, DAPT comparison, whether an LM-specific analysis was reported, and the directness of evidence for LM PCI decision-making.

### 2.2. Inclusion and Exclusion Criteria

Studies were eligible if they evaluated antiplatelet strategies relevant to patients undergoing PCI of the left main coronary artery and met at least one of the following evidence categories: (1) direct LM-specific studies enrolling exclusively LM PCI populations; (2) broader PCI studies reporting a prespecified, extractable, or clinically interpretable LM subgroup; or (3) indirect evidence from contemporary PCI, ACS, de-escalation, imaging, or revascularization trials that did not report LM-specific outcomes but informed the broader antiplatelet framework within which LM PCI decisions are made. Direct LM-specific evidence was prioritized throughout the synthesis. Indirect evidence was retained only to contextualize current practice, guideline statements, and mechanistic plausibility, and was not interpreted as equivalent to dedicated LM evidence. For all subsequent analyses, these three categories are explicitly labeled as “LM-specific”, “LM subgroup”, or “indirect contextual evidence”, and the level of directness is reported for each study and for each recommendation throughout the results and conclusion, Studies were eligible if they met all of the following criteria: (1) adult patients (≥18 years) undergoing PCI of the left main coronary artery; (2) comparison of different DAPT durations (short-term ≤6 months vs. standard 6–12 months, or extended >12 months), or evaluation of risk-score-personalized regimens; (3) reporting of clinical outcomes including all-cause mortality, major adverse cardiac events, bleeding, stent thrombosis, myocardial infarction, or repeat revascularization; (4) randomized controlled trials, prospective or retrospective cohorts, pooled patient-level analyses, or registry analyses; (5) full-text publication in English; (6) studies published from January 2010 onwards, reflecting contemporary stent technology and procedural practice; and (7) clearly defined DAPT duration with adequate follow-up (≥6 months for safety endpoints and ≥12 months for ischemic endpoints, except where otherwise specified).

Exclusion criteria comprised: (1) studies focused exclusively on non–left-main coronary interventions without an LM-specific subgroup; (2) abstracts without full-text availability or conference proceedings only (except for pivotal late-breaking trials whose full publications were subsequently retrieved); (3) case reports, editorials, narrative reviews, expert opinions or letters; (4) studies combining DAPT duration with other co-interventions without an LM-specific subgroup analysis; (5) pediatric populations (<18 years); (6) studies with unclear DAPT duration or incomplete endpoint reporting; (7) animal, in vitro or preclinical research; and (8) duplicate publications or overlapping cohorts.

In instances of overlapping cohorts or multiple publications derived from the same registry or randomized trial, we applied a predefined hierarchy to avoid double counting. When overlap was identified, we preferentially included the most comprehensive or most recent report (based on sample size and follow-up duration), ensuring that each patient population contributed only once to the qualitative synthesis. Earlier or secondary analyses from the same dataset were retained only if they provided unique subgroup information (e.g., left main–specific analyses) not available in the primary publication, and this was clearly indicated in the text. Because the direct LM-specific evidence base remains limited, some conclusions necessarily draw on broader PCI populations; these extrapolations are identified explicitly throughout the manuscript.

### 2.3. Study Design, Review Type, and Risk of Bias

This work is best characterized as a narrative review with a structured literature search, rather than a fully systematic review. The review employed predefined eligibility criteria, multiple databases, independent screening, and a PRISMA-style flow diagram to enhance transparency, but it was not prospectively registered (e.g., in PROSPERO) and did not adhere to all PRISMA requirements. No quantitative meta-analysis was undertaken due to substantial heterogeneity in study designs, DAPT duration cutoffs, endpoint definitions, and follow-up schedules across the included literature. Because the synthesis was qualitative and no quantitative pooling was performed, no dedicated statistical analysis software was used; evidence was tabulated and summarized descriptively.

Risk of bias for randomized trials was assessed qualitatively using the Cochrane RoB 2 assessment tool (Cochrane, London, UK; https://www.riskofbias.info, accessed 31 May 2026), while observational studies and registries were evaluated using the ROBINS-I tool (Cochrane, London, UK; https://www.riskofbias.info, accessed 31 May 2026).

Study-level judgments focused on key domains, including confounding, participant selection, misclassification of interventions, and completeness of outcome data. Given the narrative nature of the review and the heterogeneity of designs, these assessments were synthesized descriptively to inform the weighting of evidence in the Results and Discussion sections rather than pooled quantitatively.

**Figure 1:**
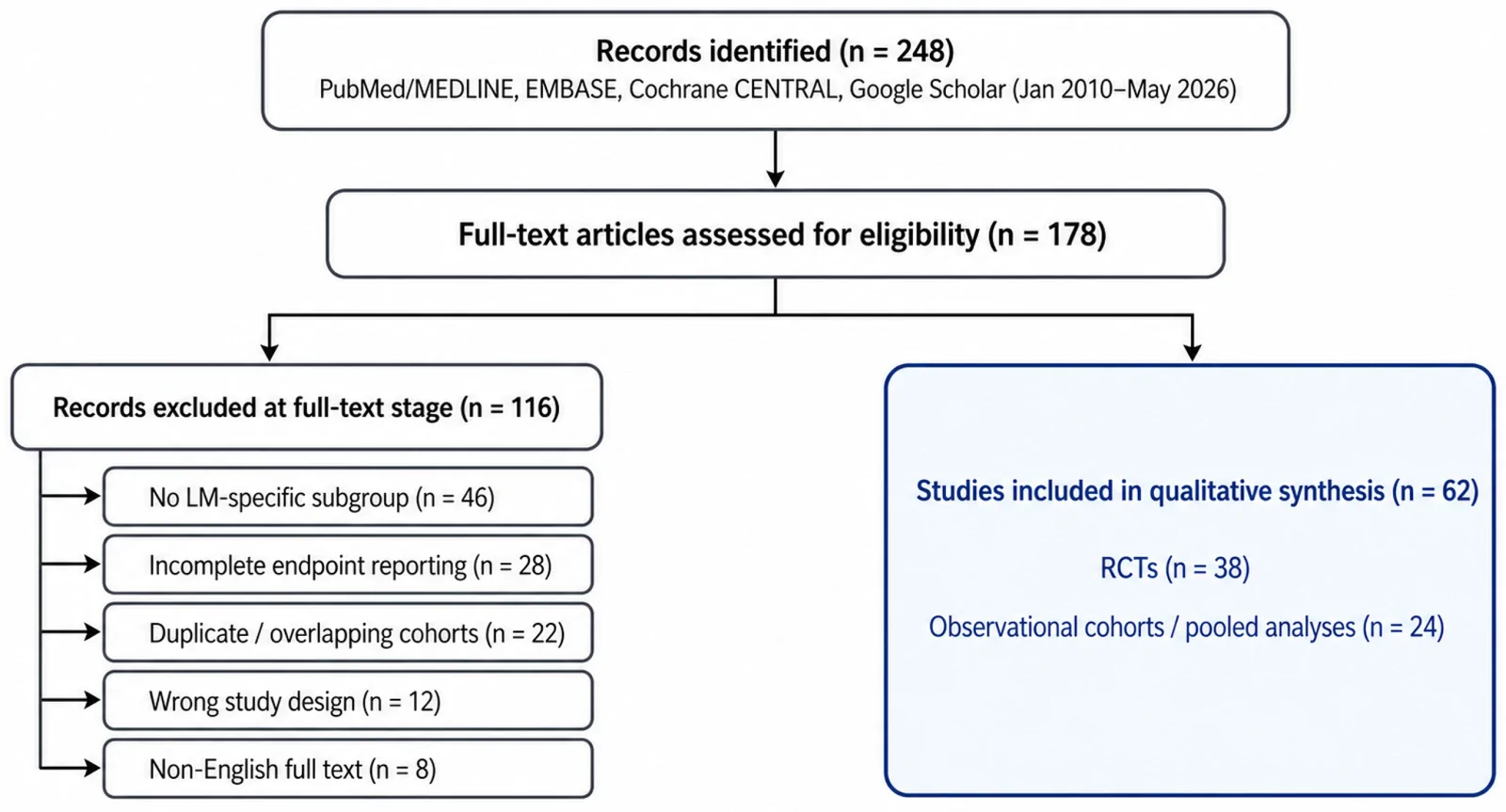
PRISMA-style flow diagram of study selection. From 248 unique records identified through PubMed/MEDLINE, EMBASE, the Cochrane Central Register of Controlled Trials, and Google Scholar (January 2010–May 2026), 178 records underwent full-text screening; 62 studies were included in the qualitative synthesis, comprising LM-specific studies, broader PCI studies with LM-subgroup data, and indirect contextual trials from non-LM PCI populations. These three evidence strata were maintained throughout the narrative synthesis and in the proposed algorithm to distinguish direct LM evidence from extrapolated data.

The modified PRISMA-style flow diagram is presented in Figure 1 (January 2010–May 2026) and was created with BioRender (BioRender, Toronto, ON, Canada; https://www.biorender.com, accessed 15 June 2026).

## 3. Guideline Recommendations

Recommendations on DAPT duration after LM PCI draw on randomized trials, observational cohorts, and meta-analyses, all of which seek to balance ischemic protection against bleeding risk. Most contemporary documents endorse at least 6 months of DAPT for stable patients receiving LM stents, based on extrapolations from broader PCI populations and supported by LM-specific registry data. Retrospective and registry-based analyses suggest that a 6-month regimen is generally sufficient in low-risk patients with stable disease, without a measurable excess of MACE compared with longer durations.

For patients with chronic coronary syndrome (CCS) undergoing PCI with drug-eluting stent implantation in the LM, the 2024 European Society of Cardiology (ESC) guideline on the management of chronic coronary syndromes recommends DAPT with aspirin and clopidogrel for 6 months as the default, provided bleeding risk is not high. A shorter window (1–3 months) may be considered in patients meeting high bleeding risk (HBR) criteria. In patients with elevated thrombotic risk—complex LM stenting, two-stent bifurcation techniques, or additional clinical risk factors—prolonged DAPT (>6 months) can be considered, although the trade-off against bleeding must be made explicit [15].

In acute coronary syndrome (ACS), the 2023 ESC guidelines [16] recommend 12 months of DAPT after LM stenting unless the patient is at high bleeding risk. Aspirin should be combined with a potent P2Y12 inhibitor—ticagrelor or prasugrel—preferred over clopidogrel, except where contraindicated; the 2023 ESC guideline notably favors prasugrel over ticagrelor in ACS patients undergoing PCI, based on ISAR-REACT 5 [17] data. Shortening DAPT in LM ACS is reserved for situations where bleeding risk is judged prohibitive, and even then, individualization is paramount, particularly as comorbidities evolve over follow-up.

The 2025 ACC/AHA/ACEP/NAEMSP/SCAI guideline [18] likewise endorses at least 12 months of DAPT after LM stenting in most patients—especially in ACS—with individual tailoring for bleeding risk and procedural complexity. Routinely extending beyond 12 months is discouraged unless ischemic risk remains high and bleeding risk low, since prolonged therapy increases hemorrhagic events without proportionate ischemic benefit in unselected populations. Both documents now acknowledge a Class IIa indication for P2Y12 inhibitor monotherapy after 1–3 months of DAPT in patients at high bleeding risk who tolerate the initial dual-therapy phase, drawing on the TWILIGHT [19], MASTER DAPT [20], TICO [21] and T-PASS [22] trials.

A concise comparison of the three most influential contemporary documents is provided in Table 1. In Table 1, formal guideline statements are presented verbatim or in close paraphrase in a dedicated column, while LM-specific interpretations and extrapolations are explicitly segregated in a separate column. This distinction is maintained throughout the manuscript so that readers can clearly differentiate between guideline-endorsed recommendations and author-derived LM-focused inferences from broader PCI and ACS evidence.

### Rationale Behind ESC Recommendations

The ESC framework leans toward a tailored DAPT duration after LM PCI in CCS, with the net clinical benefit defined as the absolute reduction in ischemic events minus the absolute increase in bleeding events. Ischemic risk—the likelihood of myocardial infarction (MI), stent thrombosis or cardiovascular death after LM PCI—is amplified by the territory at stake and is driven jointly by anatomy, comorbidities and procedural factors. Recognizing and quantifying these risks is essential to optimize both therapy duration and long-term outcomes.

Several anatomical features of the LM are associated with elevated post-stenting risk. Distal LM involvement that incorporates the ostia of both the left anterior descending (LAD) and the left circumflex (LCx) arteries—classified as a true bifurcation lesion (Medina 1/1/1)—differs markedly in complexity from isolated ostial or mid-shaft disease. Two-stent strategies such as DK-crush, culotte, and T-stenting are technically demanding and carry a higher risk of stent thrombosis and restenosis.

Long or overlapping stents increase the probability of incomplete coverage or malposition. Heavy calcification can hinder optimal stent expansion and apposition, and a substantial diameter mismatch between the LM and its branches can result in suboptimal sizing. Most stent thromboses occur within the first 30 days—a window classically termed “early stent thrombosis” [23].

## 4. Determinants of Drug-Eluting Stent Thrombogenicity

The thrombogenicity of contemporary DES reflects the interaction among device, patient, procedural, and pharmacological variables. Understanding their relative contributions is central to limiting stent thrombosis after PCI.

### 4.1. Stent Design and Material

Stent design and material influence both the safety and the optimal duration of DAPT after LM stenting. Modern drug-eluting platforms, especially those with biodegradable or polymer-free coatings, exhibit improved thromboresistance and faster endothelialization, opening the door to shorter DAPT in selected patients without increased stent thrombosis. For anatomically complex LM lesions—particularly those requiring bifurcation strategies—the residual thrombotic risk often supports prolonged DAPT (despite the bleeding penalty.

Strut thickness is a key determinant: thicker struts disrupt laminar flow and delay endothelial coverage, both of which favor thrombus formation. Stent geometry and surface finish modulate platelet activation, while alloy composition can trigger varying degrees of inflammation or hypersensitivity [24]. Ultrathin-strut platforms may further promote endothelial healing and, theoretically, support shorter DAPT durations, although robust LM-specific data remain limited. Stents with higher radial force or specialized geometries are sometimes preferred for challenging LM morphologies but have not yet altered duration recommendations, as dedicated trials are lacking [25].

The biocompatibility of polymer coatings is equally important. Non-biocompatible or durable polymers can sustain chronic inflammation, hypersensitivity, and delayed healing, whereas polymer-free or biodegradable designs may reduce long-term thrombogenicity [26]. Antiproliferative agents such as sirolimus and paclitaxel suppress smooth muscle proliferation but also impair endothelial regeneration, prolonging the risk window for thrombosis; burst release or uneven drug distribution can amplify local toxicity.

### 4.2. Procedural Factors

Two-stent strategies for LM bifurcations—double-kissing crush, culotte or T-stenting—carry a higher risk of thrombotic complications than simpler single-stent (provisional) approaches. Notably, DAPT durations shorter than 12 months increase the risk of target lesion failure and thrombotic events after two-stent LM bifurcation PCI, although they do not appear to do so after a one-stent strategy [27,28]. Greater lesion complexity—multiple stents, long lesions, heavy calcification, diffuse disease burden—increases ischemic risk and reinforces the case for longer DAPT [29].

The choice between a provisional single-stent and an upfront two-stent strategy in true distal LM bifurcations remains contested. The DKCRUSH-V [30] trial reported lower 3-year target lesion failure with double-kissing crush (8.3% vs. 16.9% with provisional stenting), whereas the European Bifurcation Club EBC MAIN [31] trial in 467 patients with true distal LM bifurcation found no significant difference in MACE at 12 months between stepwise provisional and systematic two-stent approaches, with the provisional strategy requiring a second stent in only 22% of cases. The DEFINITION II [32] trial extended these findings to complex bifurcations more broadly. Contemporary expert consensus increasingly advocates a stepwise provisional approach as the default in most LM bifurcations, reserving upfront two-stent techniques—particularly DK-crush—for complex anatomies with significant disease in the side branch (length ≥ 10 mm). Regardless of the strategy chosen, two-stent procedures uniformly mandate at least 12 months of DAPT in the absence of prohibitive bleeding risk.

Stent under expansion or malposition, by interrupting wall contact, fosters thrombus formation. Overlapping or excessively long stents expand the polymer and metal surface area exposed to flowing blood. Geographic miss and edge dissections leave injured vessel surfaces uncovered, further amplifying thrombotic risk. Bifurcation anatomy, long or calcified lesions, and diameter mismatches between main branch and side branch likewise correlate with thrombogenesis.

### 4.3. Patient-Related Factors

Prior MI, prior stent thrombosis, and prior revascularization (PCI or CABG) are well-established predictors of ischemic complications and favor longer DAPT [33]. Comorbid diabetes, renal dysfunction, reduced ejection fraction, and ACS at presentation increase thrombotic susceptibility, as do prothrombotic states such as active malignancy or systemic inflammation. Anatomical features—small vessel caliber, diffuse disease, and positive remodeling—also contribute [11].

### 4.4. Pharmacological Factors

Premature discontinuation or interruption of DAPT is the single most consistent driver of stent thrombosis. High on-treatment platelet reactivity—often linked to CYP2C19 loss-of-function variants—reduces the protective effect of clopidogrel and identifies patients in whom switching to a more potent agent should be considered.

Antiplatelet resistance—an attenuated or absent response to aspirin or clopidogrel—is more commonly reported with clopidogrel, particularly in carriers of polymorphisms that impair metabolic activation. Light transmission aggregometry and point-of-care platelet function assays can flag poor responders, but no universally accepted laboratory standard exists [34,35]. Reported prevalence varies widely (5–50% depending on the drug, population, and assay) [36]. Practical responses include switching agents, increasing the dose, or pursuing combination therapy, but their effectiveness and safety have not been fully established. Genetic testing for CYP2C19 variants can help identify poor metabolizers and inform agent selection [37]. Clinically, antiplatelet resistance is a recognized driver of thrombotic events and reflects genetic, pharmacological and patient-related factors—a persistent challenge in cardiovascular therapy.

## 5. Descriptive Synthesis of Existing Studies

The literature on optimal DAPT duration after LM stenting spans observational cohorts, randomized trials, meta-analyses, and registry analyses. Most studies grapple explicitly with the trade-off between ischemic event reduction and bleeding risk in patients undergoing LM stenting, with particular attention to procedural complexity and patient risk profile. Geographically, large cohorts from Asia, Europe, and North America capture diverse practice patterns and degrees of guideline adherence. Read together, these data inform the increasingly individualized DAPT decisions required in contemporary practice. For clarity and consistency across the heterogeneous literature, this review classifies DAPT duration into three operational categories: short (≤6 months), standard (>6 to 12 months), and prolonged (>12 months). Because individual trials and registries variably report thresholds such as 1 month, 3 months, 6 months, 12 months, or 24 months, these categories are used throughout the manuscript as umbrella definitions for synthesis rather than as rigid trial-specific labels. Accordingly, expressions such as “at least 12 months” should generally be interpreted as falling within the upper boundary of the standard category unless therapy is explicitly extended beyond 12 months, whereas durations of 18–24 months are discussed under prolonged DAPT. For clarity and consistency across the heterogeneous literature, this review classifies DAPT duration into three operational categories: short (≤6 months), standard (>6 to 12 months), and prolonged (>12 months). Because individual trials and registries variably report thresholds such as 1, 3, 6, 12, or 24 months, these categories are used throughout the manuscript as umbrella definitions for synthesis rather than as rigid trial-specific labels.

### 5.1. Observational Studies

Recent prospective observational studies have specifically examined DAPT duration after PCI with stenting for LM disease, with the primary objective of balancing ischemic risk reduction against bleeding risk [38].

Extending DAPT beyond 12 months has been associated with a significant reduction in the 3-year primary effectiveness composite and in critical ischemic endpoints such as cardiovascular death and stent thrombosis. Safety signals were reassuring: no significant excess of major bleeding was observed compared with shorter regimens, and the benefit appeared consistent across clinical risk strata, bleeding risk categories, types of P2Y12 inhibitor and stenting strategies.

The advantages of DAPT beyond 12 months must nonetheless be weighed against individual bleeding risk. In patients who remain event-free at 12 months, extended therapy appears to confer continued ischemic protection without a meaningful safety penalty. In particularly complex contexts—such as bifurcation lesions treated with two-stent techniques—maintaining DAPT for at least 12 months is critical. Although current guidelines typically recommend 6–12 months post-stenting, evidence increasingly supports longer durations in selected patients with LM stents and low bleeding risk [38].

The Korean Multicenter Angioplasty Team (KOMATE) registry [3], with associated pooled analyses of more than 1800 patients receiving second-generation DES for LM disease, recommends maintaining DAPT for 12 to 24 months in patients without prohibitive bleeding risk. Discontinuation before 12 months was linked to a higher rate of cardiac complications, while extending therapy beyond 24 months conferred no additional safety penalty in the absence of bleeding. Treatment decisions should therefore be individualized, integrating shared decision-making, patient preferences, and comorbidity profiles.

Rhee and colleagues evaluated DAPT duration after PCI for LM bifurcation lesions, comparing 1-stent and 2-stent strategies with new-generation DES. For patients managed with a complex 2-stent strategy, DAPT of at least 12 months was crucial for mitigating ischemic events. In contrast, a 1-stent (provisional) approach derived little additional benefit from extending DAPT beyond 12 months, and therapy could be tailored more freely to patient-specific risk [28].

A retrospective analysis by Hartikainen et al. of 984 patients undergoing PCI for unprotected LM stenosis in stable angina compared 6 versus 12 months of clopidogrel-based DAPT. The 1-year primary composite of all-cause mortality, MI, and target lesion revascularization occurred in 15.2% with 6 months versus 16.3% with 12 months (*p* = 0.674), with comparable rates of stent thrombosis (0.9% vs. 0.3%; *p* = 0.224) and BARC 3,4,5 bleeding (6.0% vs. 5.8%; *p* = 0.808). The authors concluded that extending DAPT beyond 6 months in stable LM patients without high thrombotic risk is not clearly supported by the data—a position that contrasts with the more nuanced messages emerging from registry and meta-analytic evidence in more complex cohorts [12].

Treatment of coronary bifurcation lesions remains one of the most demanding domains of interventional cardiology. Second-generation DES (2G-DES) and refinements in technique have expanded the feasibility of complex PCI, yet the optimal two-stent approach—both the stenting sequence and the technical method—remains contested. The COBIS III [39] registry analyzed real-world outcomes in patients treated for bifurcation lesions with 2G-DES and dedicated two-stent strategies. Over 4.4 years of follow-up, target lesion failure (TLF) rates were comparable between provisional (MV-first) and systematic (SB-first) sequences after propensity score matching. The presence of LM bifurcation disease and shorter DAPT duration—rather than the specific stenting sequence or technique—were independent predictors of TLF. Long-term outcomes with 2G-DES were therefore dominated by operator experience, lesion complexity, and patient factors.

### 5.2. Randomized Trials and Subgroup Analyses

The IDEAL-LM [25] investigators randomized 818 patients with LM disease to a bioresorbable polymer platinum-chromium everolimus-eluting stent (BP-PtCr-EES) with 4 months of DAPT versus a durable polymer cobalt-chromium everolimus-eluting stent (DP-CoCr-EES) with 12 months. Stent thrombosis and MI rates were comparable between short and standard durations in stable patients, although the trial was underpowered for rare events.

In a prespecified analysis of the PRODIGY [40] trial, Costa and colleagues examined whether the angiographic location of disease (LM or proximal LAD) modified the effect of DAPT duration. Of 1754 participants, 953 had at least 30% luminal narrowing in the LM or proximal LAD and were randomized to 6 versus 24 months of DAPT. In the subgroup with LM or proximal LAD involvement, the 24-month regimen halved the incidence of definite, probable or possible stent thrombosis (2.8% vs. 5.6%; HR 0.45, 95% CI 0.23–0.89, *p* = 0.02). No such protection was apparent outside this anatomic subgroup, and the interaction between location and DAPT duration was highly significant (*p* interaction = 0.002), independently of whether the index PCI was performed in the LM/proximal LAD. The authors concluded that LM or proximal LAD disease identifies high-risk patients who benefit from DAPT beyond 6 months, with a clear reduction in stent thrombosis and no signal of harm—an anatomic marker that should be incorporated into duration algorithms.

#### 5.2.1. The PARTHENOPE Trial: A New Paradigm for Risk-Score-Personalized DAPT

**Figure 2:**
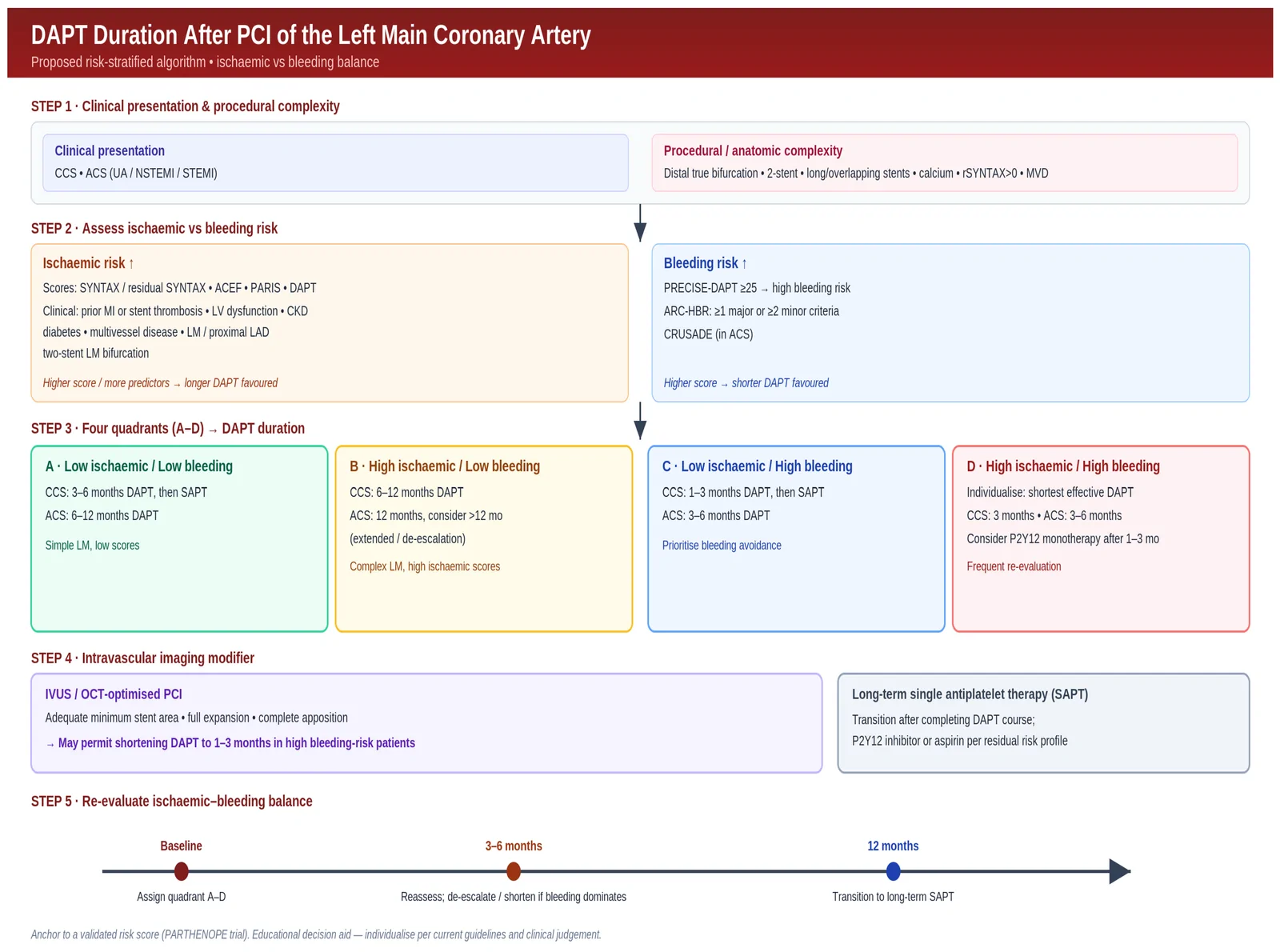
Proposed expert-opinion framework for individualized DAPT after LM PCI. Patients are first stratified by clinical presentation (CCS vs. ACS) and by procedural/anatomic complexity (distal true bifurcation, two-stent strategy, long or overlapping stents, heavy calcification, residual SYNTAX > 0, multivessel disease). Ischaemic risk is then assessed using SYNTAX/residual SYNTAX, ACEF, PARIS and DAPT scores together with clinical predictors (prior MI or stent thrombosis, left ventricular dysfunction, chronic kidney disease, diabetes, multivessel disease, LM/proximal LAD involvement, two-stent LM bifurcation). Bleeding risk is graded using PRECISE-DAPT (≥25 indicating high risk), ARC-HBR criteria (≥1 major or ≥2 minor) and—in ACS—CRUSADE. The resulting four quadrants (A–D) yield differentiated recommendations on DAPT duration in CCS and ACS. IVUS- or OCT-optimized PCI may support consideration of abbreviated DAPT in selected high-bleeding-risk patients, but imaging findings alone should not determine DAPT duration. Re-evaluation of the ischemic–bleeding balance at 3–6 and 12 months guides the transition to long-term single antiplatelet therapy (SAPT). This framework is hypothesis-generating, has not been prospectively validated in LM-specific cohorts, and incorporates both direct LM data and indirect extrapolation from broader PCI populations. The proposed framework should be interpreted as an expert opinion tool to structure individualized decision-making, not as a validated algorithm or a substitute for formal guideline recommendations. CCS = chronic coronary syndrome; ACS = acute coronary syndrome; DAPT = dual antiplatelet therapy; SAPT = single antiplatelet therapy; LM = left main; PCI = percutaneous coronary intervention; ARC-HBR = Academic Research Consortium for High Bleeding Risk; IVUS = intravascular ultrasound; OCT = optical coherence tomography.

The PARTHENOPE [6] trial, presented at ESC Congress 2025 and simultaneously published in the Journal of the American College of Cardiology by Piccolo and colleagues, represents the first dedicated randomized comparison of a risk-score-personalized DAPT strategy versus a fixed 12-month regimen after PCI. From January 2020 to June 2022, 2107 patients undergoing PCI at 14 Italian centers were randomized in a 1:1 ratio to a personalized arm—in which DAPT duration was assigned at 3, 6, or 24 months based on the DAPT score and the clinical presentation (CCS vs. ACS)—or to a standard 12-month DAPT arm. The primary endpoint, a 24-month composite of net adverse clinical events (NACE) including all-cause death, MI, stroke, urgent target vessel revascularization and BARC type 2, 3 or 5 bleeding, occurred in 196 of 1055 patients (18.6%) in the personalized arm and in 232 of 1052 patients (22.2%) in the standard arm (absolute difference −3.54 percentage points; 95% CI −6.99 to −0.99; *p* = 0.040), corresponding to a relative reduction of approximately 20%. The benefit was driven principally by reductions in MI (absolute difference of −2.29 percentage points) and revascularization, without an excess of major bleeding or death. Although LM-specific subgroup analyses were limited by sample size, PARTHENOPE [6] provides the strongest randomized evidence to date that anchoring DAPT duration in a validated risk score outperforms a uniform 12-month strategy, and directly supports the conceptual framework adopted in the algorithm proposed in Figure 2.

#### 5.2.2. NEO-MINDSET and the Limits of Very Early Aspirin Discontinuation

Whereas PARTHENOPE [6] established the value of personalization, the NEO-MINDSET [41] trial, presented at ESC Congress 2025 and published in the New England Journal of Medicine by Guimarães and colleagues, has tested an even more aggressive de-escalation strategy: immediate aspirin discontinuation after PCI for ACS with continuation of P2Y12 inhibitor monotherapy alone. The investigators randomized 3410 ACS patients across 51 Brazilian centers within four days of hospitalization to either potent P2Y12 inhibitor monotherapy (ticagrelor or prasugrel) or to conventional DAPT with aspirin and a potent P2Y12 inhibitor for 12 months. At 12 months, the primary ischemic composite (all-cause death, MI, stroke or urgent revascularization) occurred in 7.0% of the monotherapy arm versus 5.5% of the DAPT arm—a difference that failed to meet the prespecified non-inferiority margin (*p* = 0.11 for non-inferiority). A prespecified sub study published in JACC in 2026 confirmed that the directional disadvantage of monotherapy was consistent across both STEMI and NSTE-ACS subsets [42]. NEO-MINDSET [41] therefore provides an important counter-balance to the trend of progressively earlier aspirin withdrawal and reinforces the cautious stance of current ESC and ACC documents: in ACS—particularly when LM stenting is involved—the early thrombotic risk remains high enough that conventional dual therapy should generally be maintained for the first 1–3 months at a minimum, with monotherapy reserved for subsequent de-escalation. The T-PASS [22] trial, which randomized ACS patients to aspirin discontinuation at one month versus standard 12-month DAPT with ticagrelor, supports an intermediate position: monotherapy is safe when initiated after the high-risk early phase, but immediate post-PCI aspirin omission cannot yet be endorsed as routine practice—least of all after LM intervention.

### 5.3. Meta-Analyses

Meta-analyses of DAPT after LM PCI have begun to refine guideline recommendations by quantifying the trade-off between ischemic protection and bleeding in this high-risk population.

Braghieri and colleagues conducted a meta-analysis on optimal DAPT duration after PCI for LM disease, specifically examining whether extending DAPT beyond 6 or 12 months conferred additional benefit. Prolonged DAPT (>12 months) was associated with a significantly lower MACE rate than ≤12 months, with reductions in cardiac death, MI, and stent thrombosis. No significant excess of major bleeding was observed with longer DAPT regimens versus shorter regimens, and the benefit was consistent regardless of clinical presentation, bleeding risk profile, or procedural complexity, including bifurcation lesions [43]. Across these analyses, prolonged DAPT reduced MACE without a clear bleeding penalty, and the benefit applied to both stable and acute presentations and to strata of patient and lesion complexity. The cumulative weight of evidence supports moving away from a “one-size-fits-all” approach and toward considering DAPT beyond 12 months in patients at high ischemic risk and low bleeding risk, with explicit use of validated risk scores. For patients with complex bifurcations or multivessel disease, guidelines increasingly favor prolonged DAPT [40].

A systematic review and meta-analysis by Elliott and colleagues, drawing on 9 randomized trials, evaluated the use of extended DAPT beyond 12 months across clinically meaningful subgroups. Patients with prior MI derived the greatest net benefit—significant reductions in MI, stent thrombosis, and major adverse events, despite an increase in bleeding. By contrast, patients without prior MI showed fewer ischemic events but a potential rise in all-cause mortality with prolonged therapy, supporting a more cautious approach. ACS patients—particularly when ischemic complications were the dominant concern—stood to gain the most. Patients without diabetes and those under 75 generally achieved a more favorable benefit/harm balance, whereas patients with diabetes, those aged ≥75, and active smokers showed less definitive net benefit, requiring individualized decision-making [44].

### 5.4. Contemporary De-Escalation Trials Reshaping Post-PCI Antiplatelet Strategy

Beyond the dedicated LM literature, a parallel body of evidence has reshaped the antiplatelet field over the last six years through trials that, although enrolling predominantly non-LM lesions, have provided the conceptual scaffold for contemporary post-PCI antithrombotic strategy. Five of these—TWILIGHT [19], MASTER DAPT [20], STOPDAPT-2 ACS [45], TICO [21], and SMART-CHOICE [46]—have collectively defined the rationale for short DAPT followed by P2Y12 inhibitor monotherapy, while OPTION [47] has explored the substitution of aspirin within the dual regimen. The 2024 T-PASS [22] trial and the 2025 NEO-MINDSET [41] and PARTHENOPE [6] trials have most recently extended this evidence. Together with the 10-year revascularization outcomes of EXCEL [7] and NOBLE [8,9], these data delineate the framework within which any modern DAPT decision after LM PCI is now made.

The TWILIGHT [19] trial enrolled 7119 patients at high ischemic or bleeding risk who had completed three months of DAPT with ticagrelor plus aspirin after PCI with drug-eluting stents and remained event-free, then randomized them in a double-blind fashion to continue ticagrelor with aspirin or to switch to ticagrelor plus placebo for an additional twelve months. Mehran and colleagues reported a substantial reduction in BARC type 2, 3 or 5 bleeding (4.0% vs. 7.1%; hazard ratio 0.56; 95% CI 0.45–0.68; *p* < 0.001) without an increase in death, MI or stroke (3.9% in both arms; HR 0.99; 95% CI 0.78–1.25). Although the trial did not specifically enrich for LM disease, approximately one-third of enrolled lesions were anatomically complex, and a prespecified subgroup analysis suggested a consistent treatment effect in patients undergoing multivessel or complex PCI. These findings have provided the strongest randomized signal supporting de-escalation to ticagrelor monotherapy at three months in patients who tolerated short-term dual therapy.

A complementary line of evidence in high-bleeding-risk populations was provided by the MASTER DAPT [20] trial, in which 4434 patients implanted with a biodegradable-polymer sirolimus-eluting stent were randomized at one month—provided they were event-free—to either immediate discontinuation of dual therapy or its prolongation for at least two additional months. Valgimigli and co-investigators showed that abbreviated DAPT met non-inferiority for the co-primary net adverse clinical events endpoint (7.5% vs. 7.7%; difference −0.23 percentage points; 95% CI −1.80 to 1.33; *p* < 0.001 for non-inferiority) and was superior for major or clinically relevant non-major bleeding (6.5% vs. 9.4%; difference −2.82 percentage points; 95% CI −4.40 to −1.24; *p* < 0.001). Major adverse cardiac and cerebrovascular events were similar between groups (6.1% vs. 5.9%). Although patients with planned staged or LM procedures were excluded, MASTER DAPT [20] remains the largest randomized trial supporting an extremely short DAPT course in high bleeding risk individuals after contemporary DES implantation, and its findings are frequently extrapolated—with caution—to bifurcation and LM subgroups when bleeding considerations dominate.

An important counterbalance to the trend of abbreviating DAPT was supplied by the STOPDAPT-2 ACS [45] investigators, who randomized 4169 patients with acute coronary syndrome to either one-to-two months of dual therapy followed by clopidogrel monotherapy or to a conventional twelve-month aspirin-plus-clopidogrel regimen. In contrast to the parent STOPDAPT-2 [48] trial conducted in chronic coronary disease, the ACS-specific cohort did not meet non-inferiority for the composite of cardiovascular death, MI, stroke, definite stent thrombosis and TIMI major or minor bleeding (3.2% vs. 2.8%; HR 1.14; 95% CI 0.80–1.62; *p* for non-inferiority = 0.06), driven by a numerical excess of MI in the short-DAPT arm (1.6% vs. 0.9%). Major and minor bleeding were nonetheless reduced (0.5% vs. 1.2%; HR 0.46; 95% CI 0.23–0.94). These data have been frequently cited in support of preserving at least three to six months of effective platelet inhibition after ACS—particularly when the index intervention involves a high-risk site such as the LM—and they directly inform the cautious recommendations of the current ESC and ACC documents regarding ultra-short DAPT in this setting.

In a population restricted entirely to acute coronary syndrome, the TICO [21] trial randomized 3056 patients treated with ultrathin-strut sirolimus-eluting stents at 38 Korean centers to either ticagrelor monotherapy after three months of dual therapy or to a conventional twelve-month ticagrelor-based regimen. Kim and colleagues reported that the primary one-year composite of major bleeding and major adverse cardiac and cerebrovascular events was significantly lower in the short-DAPT arm (3.9% vs. 5.9%; absolute difference −1.98 percentage points; 95% CI −3.50 to −0.45; hazard ratio 0.66; 95% CI 0.48–0.92; *p* = 0.01). Major bleeding alone was also reduced (1.7% vs. 3.0%; HR 0.56; 95% CI 0.34–0.91; *p* = 0.02), while the ischemic component did not differ significantly between groups (2.3% vs. 3.4%; *p* = 0.09). TICO [21] thus complements TWILIGHT [19] by extending the evidence base for ticagrelor monotherapy at three months into an entirely ACS-treated population, although patients with isolated LM disease were under-represented and translation to anatomically complex LM bifurcation interventions remains exploratory.

Among the broader family of monotherapy de-escalation studies, SMART-CHOICE [46] randomized 2993 unselected PCI patients (chronic coronary syndrome in approximately 42% and acute coronary syndrome in approximately 58%) to three months of dual therapy followed by P2Y12 inhibitor monotherapy or to twelve months of conventional dual therapy. Hahn and colleagues observed non-inferiority for the primary one-year endpoint of all-cause death, MI or stroke (2.9% vs. 2.5%; difference 0.4 percentage points; 1-sided 95% CI upper bound 1.3%; *p* for non-inferiority = 0.007), with no significant difference in stent thrombosis (0.2% vs. 0.1%) and a meaningful reduction in BARC 2–5 bleeding (2.0% vs. 3.4%; HR 0.58; 95% CI 0.36–0.92; *p* = 0.02). The trial used clopidogrel in the majority of patients (~77%), which somewhat limits direct extrapolation to ticagrelor-based regimens but strengthens its real-world applicability—including, by extrapolation, in selected uncomplicated LM interventions.

The OPTION [47] trial introduced an alternative direction by replacing aspirin with indobufen within the dual-therapy backbone. Wu and colleagues randomized 4551 troponin-negative Chinese patients undergoing DES implantation to either indobufen 100 mg twice daily plus clopidogrel 75 mg daily or to conventional aspirin 100 mg daily plus clopidogrel 75 mg daily for twelve months. The composite primary endpoint at one year—comprising cardiovascular death, non-fatal MI, ischemic stroke, definite or probable stent thrombosis, and BARC type 2, 3 or 5 bleeding—was significantly lower with indobufen (4.47% vs. 6.11%; absolute difference −1.63 percentage points; HR 0.73; 95% CI 0.56–0.94; *p* = 0.015; *p* < 0.001 for non-inferiority). The benefit was driven almost entirely by a reduction in BARC type 2 bleeding (1.68% vs. 3.49%; HR 0.48; 95% CI 0.33–0.70; *p* < 0.001), with no significant difference in the ischemic components. Although indobufen is not currently licensed in most Western European markets, OPTION [47] provides proof-of-concept that the antiplatelet component of dual therapy can be modulated beyond the binary choice of P2Y12 inhibitor potency, and it is increasingly cited in discussions of personalized DAPT in patients with aspirin intolerance—a not-infrequent dilemma in the LM PCI population (Table 2).

## 6. Optimize Outcomes After LM Stenting: The Role of Intravascular Imaging

Intravascular imaging—encompassing intravascular ultrasound (IVUS) and optical coherence tomography (OCT)—has become integral to contemporary LM PCI and is increasingly framed in current ESC and ACC documents as a Class I or IIa recommendation depending on lesion complexity. Both modalities offer real-time, high-resolution insights that overcome the well-recognized angiographic blind spots of LM anatomy. Intravascular imaging improves lesion characterization and procedural optimization during LM PCI, but current evidence does not establish imaging-confirmed optimization as an independent, validated criterion for shortening DAPT duration; any such application should be viewed as hypothesis-generating. Several observational and meta-analytic studies suggest that intravascular-imaging-guided LM PCI achieves better stent expansion and may lower stent-related events, raising the possibility that some patients at high bleeding risk could safely receive shorter DAPT (e.g., 6 months); however, this concept remains hypothesis-generating because imaging-confirmed optimization has not been prospectively validated as an independent criterion for DAPT shortening in LM-specific cohorts. Current ESC and ACC documents acknowledge intravascular imaging as a procedural modifier of ischemic risk, but they do not mandate shorter DAPT on the basis of imaging results alone; in our framework, imaging-based shortening is therefore presented as a speculative option that should be restricted to carefully selected HBR patients after individualized assessment.

### 6.1. Intravascular Ultrasound (IVUS)

Real-time tomographic imaging informs accurate stent sizing, deployment, and confirmation of adequate lesion coverage and expansion. IVUS clarifies vessel size, plaque burden, and lesion length, supporting optimal sizing and full expansion while limiting under-expansion, malpositioning, and edge dissections. It also detects subtle complications—incomplete coverage, tissue prolapse—that are invisible on angiography, allowing immediate intra-procedural correction and thereby reducing future stent thrombosis and restenosis.

Kang and colleagues conducted a large multicenter study evaluating the long-term (up to 10-year) impact of IVUS-guided versus angiography-guided stenting in unprotected LM disease. IVUS guidance was associated with significantly lower adjusted risks of all-cause mortality and of the composite of death, MI or stroke at 10 years; the effect was consistent across patient characteristics, lesion complexity and procedural variables. Target vessel revascularization rates were similar, implying that the principal benefit of IVUS lies in reducing death and serious cardiovascular events rather than in lowering repeat procedures [50].

Meta-analyses and earlier studies support these findings: IVUS guidance during LM PCI is associated with reductions in all-cause mortality (OR ≈ 0.57), cardiovascular mortality, MI, and revascularization, with no clear effect on stroke or stent thrombosis [51,52]. Both randomized and observational data point to improved long-term prognosis, including lower hazard ratios for all-cause death and MI after adjustment for baseline risk [51]. IVUS should therefore be regarded as standard of care during LM PCI, except where contraindicated or technically infeasible, with consistent benefit across simple and complex lesions; its most enduring effect is on sustained mortality reduction over decade-long follow-up.

### 6.2. Optical Coherence Tomography (OCT) and the OCTOBER and ILUMIEN IV Trials

OCT, with axial resolution of 10–20 μm—approximately ten-fold better than IVUS—offers detailed visualization of stent strut apposition, edge dissections, plaque protrusion and neointimal coverage. Until recently, robust randomized evidence supporting OCT in LM PCI was lacking; this has changed with the publication of two pivotal trials presented at the European Society of Cardiology Congress 2023 [13,14].

The OCTOBER [13] trial, conducted by Holm and colleagues at 38 European centers, randomized 1201 patients with complex bifurcation lesions to OCT-guided PCI or angiography-guided PCI. Notably, 18.5% of patients in the OCT arm and 19.3% in the angiography arm had bifurcation lesions involving the LM coronary artery. At two years, the primary composite endpoint of cardiac death, target lesion MI, or ischemia-driven target lesion revascularization occurred in 10.1% of the OCT-guided group versus 14.1% of the angiography-guided group (hazard ratio 0.70; 95% CI 0.50–0.98; *p* = 0.035). The result was consistent across the LM subgroup, although the trial was not powered for definitive LM-specific conclusions. OCTOBER [13] provides the strongest randomized evidence to date that routine OCT guidance improves clinical outcomes in complex bifurcation PCI, including the LM.

In contrast, the parallel ILUMIEN IV [14] trial, which randomized 2487 patients with medication-treated diabetes or complex lesions to OCT-guided or angiography-guided PCI, demonstrated a larger minimum stent area and a nearly two-thirds reduction in stent thrombosis with OCT, but did not meet its primary clinical endpoint of target vessel failure at 2 years. The discordance between OCTOBER [13] and ILUMIEN IV [14] likely reflects differences in patient selection—OCTOBER [13] specifically targeted complex bifurcations, where the procedural insight afforded by OCT translates more readily into clinical benefit—and underscores that the value of OCT is greatest in anatomically challenging lesions, including the distal LM bifurcation.

Single-center, high-volume registry data from contemporary LM-PCI cohorts likewise suggest a trend toward improved survival with OCT-guided LM PCI compared with angiographic guidance, with a borderline 63% adjusted reduction in mortality (HR 0.37; *p* = 0.063) in one Romanian cohort [53]. Recognized limitations of OCT in LM PCI include the need for contrast flushing, which can be problematic in ostial or aorto-ostial lesions and in patients with renal impairment, and reduced penetration in heavily calcified vessels. In practice, IVUS remains preferred for ostial LM lesions and patients with renal dysfunction, whereas OCT is increasingly favored in distal LM bifurcations and complex stenting strategies, with IVUS as a viable alternative when OCT is technically infeasible.

### 6.3. Implications for DAPT Duration

Several observational and meta-analytic studies suggest that intravascular-imaging-guided LM PCI achieves better stent expansion and lowers stent-related events, enabling some patients at high bleeding risk to use shorter DAPT (e.g., 6 months) safely. The current ESC and ACC documents now formally acknowledge intravascular imaging as a procedural modifier that, in conjunction with risk scores, can support shortened DAPT in carefully selected HBR patients. The ultimate decision continues to depend on the balance of ischemic and bleeding risk, considering age, comorbidity, stenting technique and lesion characteristics.

## 7. Ticagrelor, Prasugrel and Clopidogrel in LM Coronary Stenting

LM coronary artery disease conveys a substantial risk owing to distinct anatomy and the extensive myocardial territory at stake, which makes selection of the oral P2Y12 inhibitor after stenting an unusually consequential decision.

Yan and colleagues [54] directly compared ticagrelor and clopidogrel in patients undergoing LM stenting. Ticagrelor was associated with a lower 3-year all-cause mortality compared with clopidogrel (5.7% vs. 8.5%; HR 0.728; 95% CI 0.537–0.985; *p* = 0.040), without a significant difference in major bleeding. Independent predictors of mortality included age, heart rate, diabetes, prior MI, low hemoglobin, elevated serum creatinine, use of ticagrelor, DAPT duration, true LM bifurcation, stent size, and use of intravascular imaging.

Prasugrel, a potent third-generation thienopyridine P2Y12 inhibitor, is recommended as part of DAPT after PCI—including LM intervention—because of the high ischemic risk inherent to this vessel. Compared with clopidogrel, prasugrel delivers more rapid, more potent, and more consistent platelet inhibition, translating into less variability in response and a lower probability of stent thrombosis [55]. In ACS, prasugrel reduces MACE more effectively than clopidogrel, particularly in high-risk anatomic subsets such as the LM and proximal LAD. In complex PCI cohorts—many of which include LM stenting—prasugrel has shown greater benefit in reducing vascular events, especially in multivessel and bifurcation interventions [56,57]. Bleeding risk requires careful assessment, given that prasugrel can increase bleeding in elderly patients, those with low body weight, and patients with prior stroke; dose adjustment or alternative agents may be needed in these populations [58]. Both European and American guidelines prefer prasugrel or ticagrelor over clopidogrel for ACS patients undergoing LM PCI, except in the presence of contraindications such as prior stroke or high bleeding risk; for stable, low-risk patients, clopidogrel remains an option.

Recent comparative studies and meta-analyses in ACS patients suggest that prasugrel may have a modest edge over ticagrelor in reducing MACE, including all-cause mortality, non-fatal MI and stroke, particularly in those managed invasively—LM stenting included [59]. The landmark ISAR-REACT 5 [17] trial found prasugrel superior to ticagrelor for the composite of death, MI or stroke, without a clear increase in major bleeding. Cohort studies and real-world registries support these findings, with the largest absolute differences observed in STEMI populations and complex cases [60]. Major bleeding rates between prasugrel and ticagrelor are broadly similar in randomized and observational settings, although both are higher than with clopidogrel [61]. Most direct head-to-head data come from ACS cohorts that include LM interventions, with relatively limited LM-specific evidence. The 2023 ESC ACS guideline, on the basis of the ISAR-REACT 5 [17] data, recommends prasugrel preferentially over ticagrelor in ACS patients undergoing PCI (Class IIa) [16]; the 2025 ACC/AHA document maintains a more even-handed position, recommending either agent.

Prasugrel is contraindicated in patients with prior stroke or transient ischemic attack, and caution is advised in those over 75 years or under 60 kg. Ticagrelor is preferred where prasugrel is contraindicated or when reversibility is required. Clopidogrel is reserved for patients in whom prasugrel and ticagrelor are contraindicated, not tolerated or unavailable, and for stable coronary disease or elective PCI with lower ischemic risk.

For LM interventions, therapy should be personalized but both potent agents remain acceptable first-line choices for most ACS patients [60].

### DAPT De-Escalation

DAPT de-escalation refers to a stepwise reduction in antiplatelet intensity after an initial high-intensity phase (typically aspirin with ticagrelor or prasugrel) and is increasingly used to minimize bleeding while preserving ischemic protection. In trials such as TROPICAL-ACS [62] and TALOS-AMI [63], de-escalation reduced bleeding without an increase in ischemic events in stabilized ACS patients, including those with complex PCI features. A de-escalation strategy—particularly when guided—appears safe once the highest-risk early period has passed (typically after 1–3 months post-PCI), and may be considered especially in patients at increased bleeding risk or with minor bleeding events, after an individualized assessment [63,64]. After LM stenting, de-escalation is increasingly accepted when tailored to patient risk and timed after the initial 1–3 months of high thrombotic risk; options include switching to clopidogrel, reducing the dose of a potent P2Y12 inhibitor, or eventually discontinuing one antiplatelet agent. Laboratory-guided assessment is particularly valuable for ensuring ongoing safety [65]. As noted above, the NEO-MINDSET [41] trial demonstrates that very early aspirin discontinuation (within four days of PCI) cannot yet be recommended in ACS, whereas the T-PASS [22] trial supports aspirin withdrawal at one month with continuation of ticagrelor monotherapy as a viable strategy in selected ACS patients [22,41].

## 8. Regional, Genetic, and Economic Influences on DAPT Decisions

### 8.1. Regional Practice Patterns

DAPT duration and procedural approaches for LM PCI differ markedly by region. Korean registry data, for example, show wide variability in post-LM DAPT duration—ranging from less than 6 months to more than 24 months—reflecting the combined influence of local guidelines, operator preference, and healthcare infrastructure [66]. European centers more commonly use radial access, whereas femoral access remains dominant across parts of the Americas and Asia/Pacific. Bifurcation strategy choices likewise vary regionally: double-kissing crush is increasingly common globally, while culotte stenting remains more prevalent in parts of Europe [67]. The relative uptake of ESC and ACC/AHA guidelines also shapes DAPT duration and agent selection [10].

ESC guidelines often recommend 6 months of DAPT in stable patients, with shorter durations considered in those at high bleeding risk and prolongation beyond 6–12 months reserved for high-risk anatomic or procedural scenarios. American guidelines typically default to 12 months, with flexibility for adjustment based on patient-specific risk. East Asian practice—captured by registries in Korea and Japan—reflects greater willingness to individualize DAPT duration based on post-procedural risk, but also notable caution driven by bleeding concerns and the prevalence of genetic factors affecting antiplatelet metabolism.

### 8.2. Genetic Polymorphisms

Polymorphisms in CYP2C19 are the most clinically relevant genetic determinants of clopidogrel response. Loss-of-function variants (CYP2C19*2 and *3), more prevalent in East Asian populations, reduce the conversion of clopidogrel to its active metabolite and predispose to stent thrombosis and recurrent MI [68]. Additional genes (PEAR-1, CES1, PON1, P2Y12) further modulate response, although their clinical impact is less consistent. Some Western centers now apply genotype-guided agent selection (preferring ticagrelor or prasugrel in poor metabolizers), but this remains far from routine and depends on resources, regulatory environment, and clinician familiarity.

### 8.3. Economic Factors

Economic considerations influence both drug selection and DAPT duration. Clopidogrel—being cheaper and more widely available than ticagrelor or prasugrel—is often the default in lower-resource settings, even when pharmacogenetic factors argue against it. In systems without universal healthcare or with limited insurance coverage, longer DAPT can be financially unsustainable, leading to premature discontinuation or use of suboptimal regimens. In well-resourced systems, guideline-concordant durations and modern P2Y12 inhibitors are more common. Although prolonged DAPT reduces ischemic events, the associated bleeding risk and incremental drug costs require a nuanced assessment of patient risk and economic realities at both individual and system levels [38].

## 9. Evaluation of Ischemic and Bleeding Risk After LM PCI

### 9.1. Ischaemic Risk

Estimating ischemic risk after LM PCI requires integrating patient comorbidities, procedural complexity, angiographic findings, and validated risk scores to guide both therapy selection and prognostication [69].

In practice, this typically involves four steps: (1) collecting clinical data—age, comorbidities, LVEF, prior events, kidney function; (2) reviewing procedural details—stent complexity, total stent length, lesion characteristics, completeness of revascularization; (3) calculating risk scores—SYNTAX, ACEF, PARIS and DAPT; and (4) identifying high-risk features.

The SYNTAX score [70] is calculated pre-PCI from angiographic features and quantifies the complexity and extent of coronary artery disease. Higher scores predict greater risk of ischemic events—cardiac death, MI, or repeat revascularization—with a threshold of ≥13 often used to denote elevated risk [71]. The Residual SYNTAX score (rSS) is calculated after PCI based on any remaining significant lesions; rSS > 0 indicates residual risk, with higher values correlating with increased long-term ischemic risk [71].

The ACEF (Age, Creatinine, Ejection Fraction) score, calculated as Age/LVEF (%) + 1 (if serum creatinine > 2.0 mg/dL), concisely stratifies risk; ACEF ≥ 1.0225 indicates higher risk [72]. The PARIS score integrates clinical and procedural variables—age, prior ACS, diabetes, smoking, chronic kidney disease, prior PCI/CABG, and revascularization features—to predict ischemic and bleeding events after PCI, with stratification into low-, intermediate-, or high-risk categories [73]. In the START ANTIPLATELET Registry, the ACEF score performed comparably to the more complex PARIS score for predicting ischemic and bleeding events in ACS patients, and is favored in routine practice because it requires only age, creatinine, and ejection fraction—data immediately available at the bedside [72].

The DAPT score is a validated clinical tool designed to identify which patients benefit from extending DAPT beyond 12 months rather than stopping earlier, by weighing ischemic versus bleeding risk. A score ≥ 2 suggests favorable net benefit from extended DAPT, while a score < 2 implies minimal or no net benefit and supports discontinuation. The score integrates age, diabetes, smoking, prior PCI or MI, congestive heart failure or reduced LVEF, stent characteristics (size, location, type), and vein graft stenting [74]. The PARTHENOPE [6] trial used the DAPT score as the principal anchor for its personalized arm, providing the first randomized demonstration that score-guided durations improve net clinical outcomes after PCI.

Beyond scoring systems, ESC/EACTS guidelines and validated studies define several high-risk features: implantation of ≥3 stents, bifurcation lesions requiring two stents, multivessel disease in diabetes, treatment of chronic total occlusion, and renal insufficiency.

### 9.2. Bleeding Risk

The PRECISE-DAPT score estimates the probability of bleeding in patients on DAPT after coronary stenting and is widely used to guide duration in patients with elevated hemorrhagic risk. It is derived from age, creatinine clearance, hemoglobin, white blood cell count, and prior spontaneous bleeding. The score stratifies risk into very low (≤10), low (11–17), moderate (18–24), and high (≥25) categories. A score ≥ 25 identifies patients in whom a shorter DAPT regimen is generally advisable; patients with lower scores can usually be maintained on longer therapy to maximize ischemic protection. In summary, PRECISE-DAPT is an evidence-based, validated tool that helps tailor DAPT duration to bleeding risk and improves outcomes by balancing ischemic protection against hemorrhagic harm [75].

The Academic Research Consortium for High Bleeding Risk (ARC-HBR) criteria provide a standardized definition of high bleeding risk in PCI populations, including those undergoing LM stenting. A patient is classified as HBR if they meet at least one major or two minor criteria. Major criteria include severe prior bleeding, thrombocytopenia, active malignancy, or severe renal failure; minor criteria include age ≥ 75 years, moderate anemia, and moderate renal impairment. The framework is used in both trials and routine practice to identify HBR patients and to tailor antiplatelet regimens [76].

The CRUSADE score quantifies the probability of in-hospital major bleeding in patients with NSTE-ACS, including both spontaneous and procedure-related events. Stratification into low, intermediate, or high categories supports decisions regarding antithrombotic aggressiveness and bleeding precautions and is particularly useful after LM stenting or other complex PCI to titrate DAPT duration and antithrombotic strategy in patients with elevated bleeding propensity [77].

Patients undergoing LM stenting represent a high-risk population in which the challenge is to optimize the balance between ischemic events (stent thrombosis, MI, death) and bleeding complications, both of which carry substantial mortality. Extended DAPT reduces stent thrombosis, MI, and repeat revascularization; in patients at high bleeding risk, shorter DAPT is preferable because bleeding can drive morbidity and mortality, increase antithrombotic discontinuation, and, in turn, raise ischemic risk. Potent agents such as prasugrel and ticagrelor offer superior ischemic protection at the cost of increased bleeding risk; clopidogrel may be preferred where bleeding risk predominates. Because bleeding is independently associated with increased mortality after LM stenting, minimizing it is as important as preventing ischemic events, especially in a vessel where ischemic events can themselves be fatal.

ESC and ACC/AHA guidelines therefore endorse validated risk scores—PRECISE-DAPT, ARC-HBR and the DAPT score—to actively balance ischemic and bleeding risks rather than apply a uniform approach. Patients with multiple high-risk features should be considered for extended DAPT and more frequent follow-up, with explicit attention to bleeding risk. Risk should be reassessed at every follow-up visit because the probability of major ischemic events remains highest in the first two years after LM PCI. Decisions should integrate scoring systems with clinical judgment, taking full account of each patient’s evolving risk profile.

We propose an operational summary of recommended DAPT durations across key left main clinical scenarios, integrating guideline recommendations, LM-specific registries, and extrapolated evidence from contemporary de-escalation trials. In each quadrant, we indicate both the suggested duration range and whether the supporting evidence is primarily LM-specific or derived from broader PCI cohorts (Table 3).

## 10. Knowledge Gaps and Future Directions

Despite considerable research, several methodological limitations affect the interpretation and generalizability of current evidence on DAPT duration after LM stenting, and a number of clinically important questions remain to be answered by future research.

### 10.1. Study Design and Population

Many studies are observational cohorts or registries and are therefore prone to confounding and selection bias, with duration decisions often left to physician discretion rather than randomization. Elderly patients, those with renal dysfunction, high bleeding risk or complex LM lesions (bifurcation, two-stent strategies) are frequently underrepresented, limiting applicability to these populations. Some of the largest studies are also dominated by male participants, reducing relevance for women. Another important gap is the limited representation of women in both LM-specific registries and in the broader de-escalation and personalization trials from which LM practice is often extrapolated. Because women undergoing PCI are, on average, older, more likely to have lower body weight, anemia, and a higher baseline bleeding propensity, under-enrolment weakens confidence in the external validity of current DAPT-duration algorithms for female patients with LM disease. At the same time, most available studies provide only sparse sex-stratified analyses, making it difficult to determine whether the ischemic--bleeding trade-off and the net clinical benefit of abbreviated versus prolonged DAPT differ meaningfully by sex in the LM setting. A related limitation concerns frail older adults, the very elderly (>80 years), and patients with severe chronic kidney disease, all of whom are frequently encountered in real-world LM PCI but remain underrepresented in randomized trials and even in many contemporary registries. These patients often carry both heightened thrombotic risks, because of diffuse atherosclerosis, calcification, and clinical instability, and heightened bleeding risk, because of age, comorbidity, polypharmacy, impaired drug clearance, and vulnerability to procedural complications. As a result, the populations in whom DAPT duration is most difficult to individualize are often those least directly represented in the evidence base.

### 10.2. Heterogeneity in Definitions and Protocols

In this review, DAPT duration is operationally classified as short (≤6 months), standard (>6 to 12 months), and prolonged (>12 months), in line with contemporary guideline-based and trial-based usage. Studies use inconsistent cutoffs for short-, standard-, and prolonged-DAPT, which complicates direct comparisons across trials and registries. Definitions of MACE, bleeding, and stent thrombosis vary, hampering pooled analyses and meta-analyses. The transition from first- to second-generation DES across the inclusion window adds further heterogeneity, since newer platforms may permit shorter DAPT with comparable safety and efficacy.

### 10.3. Sample Size and Statistical Power

Even in large registries, the number of patients with complex LM lesions or with specific stenting techniques (e.g., two-stent bifurcation) is often modest, limiting subgroup analyses. Many studies are also underpowered to detect rare but critical events such as stent thrombosis or fatal bleeding, particularly in LM populations.

### 10.4. Duration of Follow-Up

Follow-up windows vary and are sometimes short, missing late events such as very late stent thrombosis or post-DAPT bleeding. Loss to follow-up over time can introduce bias, especially when attrition is non-random. The recently published 10-year NOBLE [9] data illustrate both the value and the rarity of truly long-term follow-up after LM revascularization.

### 10.5. Real-World Practice and Generalisability

There is wide variation in real-world DAPT duration after LM PCI, reflecting uncertainty and the absence of standardized protocols. Few studies use validated risk scores to tailor DAPT duration, and most rely on physician judgment, which is neither uniform nor always evidence-based. The PARTHENOPE [6] trial offers a template for embedding risk-score-driven decisions into routine practice, but its findings require external validation in dedicated LM cohorts. Generalizability is also constrained by the fact that widely used bleeding-risk tools such as PRECISE-DAPT and ARC-HBR were derived and validated primarily in broader PCI populations rather than in cohorts specifically enriched for left main disease. Although these instruments are clinically useful and are appropriately incorporated into current practice, their calibration in LM-only populations remains uncertain, particularly in patients with large-territory myocardium at risk, complex bifurcation anatomy, two-stent strategies, advanced age, frailty, or severe renal dysfunction. In other words, a score that performs adequately in an all-comer PCI cohort may not fully capture the distinct balance between catastrophic ischemic consequences and major bleeding liability that characterizes LM PCI.

### 10.6. Residual Confounding

Even with statistical adjustment, unmeasured factors—frailty, socioeconomic status, medication adherence—can influence both DAPT duration and outcomes, producing residual confounding.

### 10.7. Future Directions

Several ongoing and planned research efforts will progressively reduce these uncertainties. Dedicated LM PCI registries with prospective collection of imaging, risk-score, and outcome data are needed to externally validate the PARTHENOPE [6] paradigm specifically in LM populations. Trials directly testing risk-score-guided versus uniform DAPT strategies in two-stent LM bifurcation cohorts represent the highest unmet need, given that this subgroup is consistently underrepresented in monotherapy and de-escalation trials. The role of OCT versus IVUS guidance in LM PCI warrants further head-to-head investigation, ideally with prespecified DAPT-duration co-interventions. Pharmacogenomic-guided antiplatelet strategies, having matured beyond CYP2C19 alone, are likely to become more practical as point-of-care genotyping spreads; their integration with risk-score-guided duration represents a logical next step. Finally, the contribution of artificial-intelligence-based decision-support tools—capable of integrating angiographic, intravascular-imaging, clinical, and laboratory inputs in real time—has begun to be explored and may prove particularly valuable in the high-stakes, multi-variable decision environment of LM PCI. Future studies should therefore move beyond simple extrapolation and prospectively evaluate whether existing bleeding- and ischemic-risk scores retain discrimination and calibration in LM-specific populations, and whether sex, frailty, biological age, and renal dysfunction should be incorporated more explicitly into LM-adapted decision tools. Such work would be especially valuable in patients currently at the margins of trial evidence, including women, octogenarians, patients with severe CKD, and those undergoing complex distal LM bifurcation PCI.

## 11. Limitations

This review has several limitations that should be considered when interpreting its conclusions. First, the review was not developed under a registered protocol (for instance, on PROSPERO) and, although a structured search strategy and predefined eligibility criteria were used, the process did not fully adhere to PRISMA standards, which may limit reproducibility and introduce selection bias. Second, only English-language studies published from 2010 onwards were included, potentially excluding relevant earlier or non-English reports and introducing language and publication bias.

Third, the evidence synthesized here is heterogeneous with respect to study design (RCTs, registries, observational cohorts), definitions of “short” versus “prolonged” DAPT, endpoint definitions, follow-up duration and the extent to which LM-specific data were reported separately from broader PCI populations. These differences precluded a formal meta-analysis focused solely on LM PCI and limit the ability to generate precise, quantitative effect estimates for specific DAPT strategies.

Fourth, although risk of bias was qualitatively assessed (including ROBINS-I for observational studies and the Cochrane RoB 2 framework for randomized trials), detailed risk-of-bias tables and study-level assessments are not presented, reducing transparency about the internal validity of individual studies.

Fifth, much of the LM-specific evidence on DAPT duration comes from observational registries and subgroup analyses of trials that were not designed primarily for LM disease, leaving the results vulnerable to residual confounding and indication bias despite statistical adjustment. High-risk subgroups—very elderly patients, those with severe renal dysfunction, extreme bleeding risk or highly complex bifurcation anatomy—remain underrepresented, limiting generalizability.

Sixth, even the most recent randomized data integrated into this review (PARTHENOPE [6], NEO-MINDSET [41], OCTOBER [13], ILUMIEN IV [14], T-PASS [22], NOBLE 10-year [9]) did not enrich specifically for the LM population, with the partial exception of OCTOBER (18.5% LM); their conclusions therefore require external validation in dedicated LM cohorts before becoming firmly embedded in LM-specific recommendations. Several sections necessarily extrapolate from broader ACS or complex PCI data when LM-specific evidence is sparse; recommendations regarding de-escalation, antiplatelet resistance and genotype-guided therapy should therefore be viewed as hypothesis-generating rather than definitive for LM PCI. Figure 2 and Table 3 should therefore be regarded as an unvalidated, expert-opinion framework that grades recommendations by evidence directness (LM-specific vs. subgroup vs. indirect) and is intended to support, not replace, formal guideline documents or individualized clinical judgment.

## 12. Conclusions

Compared with earlier LM DAPT narratives, the present review advances from a largely duration-based framing of “6 vs. 12 vs. >12 months” to an operational model in which DAPT duration is coupled to validated ischemic and bleeding risk scores and modified by the quality of procedural optimization documented by IVUS or OCT. Through Figure 2 and Table 3, it explicitly codifies the main left main clinical scenarios—CCS with simple LM anatomy, CCS with complex LM anatomy, ACS with simple LM anatomy, and ACS with complex LM anatomy, each further interpreted according to high-bleeding-risk status—into suggested duration ranges that are directly usable in practice. Just as importantly, the framework distinguishes recommendations grounded primarily in LM-specific evidence from those extrapolated from broader ACS, complex PCI, and de-escalation trials, thereby making transparent where current practice is evidence-based and where it remains inferential. This structure positions the review not simply as another narrative summary, but as a practical, evidence-graded decision framework for contemporary LM PCI.

The optimal duration of dual antiplatelet therapy after left main PCI cannot be reduced to a single fixed regimen; instead, it must be individualized according to clinical presentation, lesion and procedural complexity, bleeding risk, and the quality of stent implantation. In stable patients with anatomically simple LM lesions treated with contemporary drug-eluting stents and without high bleeding risk, the available LM-specific registries and guideline recommendations support 6–12 months of DAPT, with 6 months as a reasonable default and extension to 12 months in the presence of additional ischemic features. In contrast, ACS presentations and complex LM anatomy—particularly true distal bifurcations managed with two-stent strategies— generally favor at least 12 months of DAPT, and extension beyond 12 months appears beneficial in selected low-bleeding-risk patients, a recommendation grounded in LM-enriched cohorts, proximal large-territory subgroup analyses, and extended-DAPT meta-analyses. 12 months of DAPT is supported predominantly by indirect and observational evidence in ACS or complex LM PCI when bleeding risk is acceptable, although direct randomized evidence for LM-specific DAPT remains limited.

Beyond these scenarios, several strategies remain largely extrapolative. Ultra-short DAPT courses (≤3 months) followed by P2Y12 inhibitor monotherapy in LM high-bleeding-risk patients are supported mainly by de-escalation trials conducted in broader PCI populations and by imaging-guided studies with only limited LM representation; their application to LM should therefore be cautious and reserved for cases in which bleeding risk clearly predominates despite optimal stent deployment. Similarly, very early aspirin withdrawal and upfront P2Y12-only regimens after LM PCI, particularly in ACS, are informed by general ACS and complex PCI data but lack robust LM-specific validation, and recent trials caution against indiscriminate adoption of such approaches in the highest-risk subsets. The emerging paradigm, exemplified by risk-score-guided strategies such as PARTHENOPE [6], supports a shift from one-size-fits-all durations toward personalized DAPT that explicitly integrates validated scores, intravascular imaging, and procedural optimization. In left main disease, this translates into a tiered approach: high-certainty, guideline-aligned recommendations for stable, simple, and complex LM scenarios; and carefully framed, hypothesis-generating strategies for LM-HBR and aggressive de-escalation, where ongoing trials and registries are still refining the balance between ischemic protection and bleeding risk. This review synthesizes current evidence into an operational framework while highlighting where practice can be confident and where clinical judgment and shared decision-making must compensate for remaining gaps in LM-specific data.

## Figures and Tables

**Table 1:** Comparative overview of current ESC and ACC/AHA guideline recommendations relevant to DAPT duration after PCI of the left main coronary artery, with formal guideline statements clearly separated from LM-specific interpretation and author-derived extrapolation. Guideline documents rarely provide dedicated randomized-trial-based LM-specific DAPT duration recommendations; where LM-specific wording is absent, the LM interpretation shown in this table reflects the authors’ synthesis of broader guideline principles and available LM-focused studies. ACS = acute coronary syndrome; CCS = chronic coronary syndrome; DAPT = dual antiplatelet therapy; HBR = high bleeding risk; LM = left main; PCI = percutaneous coronary intervention.

Guideline	Formal Guideline Recommendation for DAPT Duration	LM-Specific Interpretation/Author Extrapolation
2023 ESC ACS [16]	Default 12 months of DAPT after ACS; shorter duration may be considered when bleeding risk is high	In LM PCI presenting as ACS, 12 months should be presented as the default guideline-based duration, while shorter regimens should be described as selective exceptions driven by bleeding risk rather than as routine LM practice
2024 ESC CCS [15]	Default 6 months of DAPT after PCI in CCS; shortening to 1–3 months may be considered in HBR patients	In stable LM PCI, 6 months may be presented as the default guideline-aligned duration, while extension toward 12 months in distal bifurcation PCI, two-stent strategies, or other high-ischemic-risk LM scenarios should be labeled as LM-focused extrapolation rather than explicit guideline wording
2025 ACC/AHA/ACEP/NAEMSP/SCAI [18]	12 months is the default after ACS; at least 6 months is generally used after CCS PCI, with shorter duration considered when bleeding risk predominates	For LM PCI, this supports individualized duration according to ischemic versus bleeding risk, but any statement favoring extension beyond 12 months in complex LM PCI should be identified as an author-derived interpretation supported mainly by observational LM data and indirect broader PCI evidence

**Table 2:** Synoptic summary of the eleven contemporary trials that frame the antiplatelet and revascularization context for left main PCI. Nine de-escalation trials (TWILIGHT [19], MASTER DAPT [20], STOPDAPT-2 ACS [45], TICO [21], SMART-CHOICE [46], T-PASS [22], OPTION [47], NEO-MINDSET [41] and PARTHENOPE [6]) are grouped with the two landmark left-main revascularization studies (EXCEL [7] and NOBLE [8,9]), the latter shown with its definitive 10-year follow-up. ACS = acute coronary syndrome; ASA = acetylsalicylic acid; BARC = Bleeding Academic Research Consortium; BP-SES = biodegradable-polymer sirolimus-eluting stent; CABG = coronary artery bypass grafting; CCS = chronic coronary syndrome; CoCr = cobalt-chromium; CRNM = clinically relevant non-major; DAPT = dual antiplatelet therapy; DES = drug-eluting stent; EES = everolimus-eluting stent; HBR = high bleeding risk; LM = left main; MACCE = major adverse cardiac and cerebrovascular events; NACE = net adverse clinical events; PCI = percutaneous coronary intervention; SAPT = single antiplatelet therapy; SES = sirolimus-eluting stent.

Trial	Year/Journal	N/Population	Intervention	Primary Endpoint	Practical Implication for LM PCI
TWILIGHT [19]	NEJM, 2019	7119 high-risk PCI [49]	Ticagrelor + ASA × 3 mo → ticagrelor monotherapy × 12 mo vs. ticagrelor + ASA × 15 mo	BARC 2/3/5 bleeding: 4.0% vs. 7.1% (HR 0.56)	Strong support for de-escalation to ticagrelor monotherapy at 3 mo; applicable to complex LM PCI
MASTER DAPT [20]	NEJM, 2021	4434 HBR with BP-SES	DAPT 1 mo → SAPT vs. DAPT ≥3 mo	NACE non-inferior; major/CRNM bleeding ↓ (6.5% vs. 9.4%)	Validates very short DAPT in HBR; extrapolable with caution to LM-HBR
STOPDAPT-2 ACS [45]	JAMA Cardiol, 2022	4169 ACS (DES CoCr)	DAPT 1–2 mo → clopidogrel monotherapy vs. DAPT 12 mo	Composite: 3.2% vs. 2.8%—non-inferiority NOT met	Caution: ultra-short DAPT in ACS may increase ischemic risk; even more relevant for LM
TICO [21]	JAMA, 2020	3056 ACS (ultrathin SES)	Ticagrelor + ASA × 3 mo → ticagrelor monotherapy vs. ticagrelor-based DAPT 12 mo	NACE: 3.9% vs. 5.9% (HR 0.66; *p* = 0.01)	Confirms TWILIGHT strategy specifically in ACS; LM population under-represented
SMART-CHOICE [46]	JAMA, 2019	2993 PCI (mixed CCS/ACS)	DAPT 3 mo → P2Y12 monotherapy vs. DAPT 12 mo	MACCE: 2.9% vs. 2.5% (non-inferior); BARC 2–5: 2.0% vs. 3.4%	Support for P2Y12 monotherapy in non-HBR; applicable to uncomplicated LM PCI
T-PASS [22]	Circulation, 2024	2850 ACS [49]	Ticagrelor + ASA < 1 mo → ticagrelor monotherapy vs. ticagrelor + ASA 12 mo	NACE: 2.8% vs. 5.2% (HR 0.54; non-inferior + superior)	Supports aspirin withdrawal at 1 mo in ACS; applicable to uncomplicated LM
OPTION [47]	Circulation, 2023	4551 troponin-negative DES (China)	Indobufen + clopidogrel vs. ASA + clopidogrel × 12 mo	Composite: 4.47% vs. 6.11% (HR 0.73; *p* = 0.015)	Alternative to ASA in intolerance; emerging relevance for LM with bleeding risk
NEO-MINDSET [41]	NEJM, 2025	3410 ACS (post-PCI)	Potent P2Y12 monotherapy from discharge vs. DAPT 12 mo	Composite: 7.0% vs. 5.5%—non-inferiority NOT met (*p* = 0.11)	Cautions against immediate aspirin withdrawal in ACS; reinforces ≥ 1–3 mo dual therapy in LM PCI
PARTHENOPE [6]	JACC, 2025	2107 PCI (mixed CCS/ACS)	Personalized DAPT (3/6/24 mo by DAPT score) vs. fixed 12 mo	NACE 24 mo: 18.6% vs. 22.2% (*p* = 0.040)	First RCT validating risk-score personalization; directly underpins individualized LM-DAPT algorithms
EXCEL [7]	NEJM, 2019 (5y)	1905 LM with SYNTAX ≤ 32	PCI with EES vs. CABG	Death/stroke/MI 5y: 22.0% vs. 19.2% (non-inferior)	Justifies PCI as option in low/intermediate LM; mandates optimal post-PCI DAPT
NOBLE [8,9] (10-y)	Lancet, 2026	1201 LM (88% biolimus-DES)	PCI vs. CABG	All-cause mortality 10y: 23% vs. 25% (HR 0.93; *p* = 0.56)	Final 10-yr data: PCI as safe as CABG for survival; favors PCI in ACS subset (HR 0.57); reinforces value of robust DAPT after PCI

**Table 3:** Suggested considerations for DAPT duration after LM PCI according to clinical scenario and evidence directness.

Clinical Scenario	Suggested DAPT Duration	Key LM-Specific Evidence	Extrapolated Evidence
CCS, simple LM anatomy (single-stent, non-bifurcation or non-true bifurcation), low bleeding risk	6–12 months of aspirin + clopidogrel; consider 6 months as default, with extension toward 12 months in presence of additional ischemic features	LM registries, KOMATE [3] supporting ≥12 months in higher-risk anatomies; Hartikainen et al. [12] showing no clear benefit of extending beyond 6 months in stable, lower-risk LM	General DES trials and CCS guideline recommendations for standard-risk PCI populations
CCS, complex LM (true distal bifurcation, two-stent strategy, long or overlapping stents), low bleeding risk	At least 12 months of DAPT; consider extension to 18–24 months in the absence of bleeding, especially after two-stent bifurcation	LM bifurcation studies and registries (e.g., Rhee et al. [28], COBIS-III [39] LM subset) showing higher event rates with shorter DAPT, PRODIGY [40] LM/proximal LAD subgroup supporting prolonged therapy	Meta-analyses of extended DAPT in complex PCI and proximal large-territory lesions
CCS, LM with high bleeding risk (ARC-HBR, PRECISE-DAPT high), anatomically optimized with IVUS/OCT	3–6 months of DAPT followed by P2Y12 monotherapy; lean toward 3 months if bleeding risk is predominant and imaging confirms optimal result	Limited LM-specific data; small LM subsets in imaging-guided registries	TWILIGHT [19], MASTER DAPT [20], TICO [21], SMART-CHOICE [46] and guideline-endorsed P2Y12-monotherapy strategies in HBR populations, extrapolated to LM with caution
ACS with LM involvement, simple anatomy (single-stent, no complex bifurcation), low bleeding risk	≥12 months of DAPT with aspirin + potent P2Y12 inhibitor (prasugrel or ticagrelor); consider extension beyond 12 months if additional high-risk features (prior MI, multivessel disease) present	LM-enriched ACS registries and PRODIGY [40] LM/proximal LAD subgroup demonstrating benefit of prolonged DAPT	ESC and ACC/AHA ACS guidelines; extended-DAPT meta-analyses showing benefit in post-MI populations
ACS with complex LM (true distal bifurcation, two-stent strategy), low bleeding risk	12 months of DAPT is generally favored in ACS or complex LM PCI when bleeding risk is acceptable, although direct LM-specific randomized evidence remains limited, extension beyond 12 months may be considered in carefully selected patients with persistent high ischemic risk and low bleeding risk, largely on the basis of observational LM data and extrapolated evidence	LM bifurcation cohorts showing increased target lesion failure and thrombosis with shorter DAPT; KOMATE [3] suggesting benefit of 12–24 months in complex LM	Extended-DAPT trials and meta-analyses in complex/ACS PCI; de-escalation trials cautioning against very early aspirin withdrawal in ACS (e.g., NEO-MINDSET [41])
ACS with LM, high bleeding risk (HBR), anatomically optimized with IVUS/OCT	6–12 months of DAPT; avoid shortening below 6 months in the absence of prohibitive bleeding; consider P2Y12 monotherapy after 1–3 months only on a case-by-case basis	Scarce LM-specific evidence; extrapolation from general ACS PCI complicated by higher LM risk	T-PASS [22] and other de-escalation trials supporting P2Y12 monotherapy after the early high-risk phase; NEO-MINDSET [41] cautioning against immediate aspirin discontinuation, extrapolated carefully to LM

CCS = chronic coronary syndrome; ACS = acute coronary syndrome; LM = left main; HBR = high bleeding risk; IVUS = intravascular ultrasound; OCT = optical coherence tomography.

## Data Availability

No new data were created or analyzed in this study. Data sharing is not applicable to this article.

## Supplementary Materials

The following supporting information can be downloaded at: https://www.mdpi.com/article/10.3390/medicina62081487/s1, Table S1: Randomized trials informing dual antiplatelet therapy (DAPT) decision-making after left main (LM) percutaneous coronary intervention: LM sample size, antiplatelet comparison, LM-specific analysis, and directness of evidence.

## Abbreviations

ACC/AHA/ACEP/NAEMSP/SCAI	American College of Cardiology/American Heart Association/American College of Emergency Physicians/National Association of EMS Physicians/Society for Cardiovascular Angiography and Interventions
ACEF	Age, Creatinine, Ejection Fraction
ACS	Acute coronary syndrome
ARC-HBR	Academic Research Consortium for High Bleeding Risk
ASA	Acetylsalicylic acid
BARC	Bleeding Academic Research Consortium
BP-PtCr-EES	Bioresorbable polymer platinum-chromium everolimus-eluting stent
BP-SES	Biodegradable-polymer sirolimus-eluting stent
CABG	Coronary artery bypass grafting
CCS	Chronic coronary syndrome
CI	Confidence interval
CoCr	Cobalt-chromium
CRNM	Clinically relevant non-major
CRUSADE	Can Rapid Risk Stratification of Unstable Angina Patients Suppress Adverse Outcomes with Early Implementation of the ACC/AHA Guidelines
CYP2C19	Cytochrome P450 2C19
DAPT	Dual antiplatelet therapy
DES	Drug-eluting stent
DK-crush	Double-kissing crush
DP-CoCr-EES	Durable polymer cobalt-chromium everolimus-eluting stent
EBC MAIN	European Bifurcation Club Left Main Coronary Stent study
EES	Everolimus-eluting stent
ESC	European Society of Cardiology
HBR	High bleeding risk
HR	Hazard ratio
ILUMIEN IV	Optical Coherence Tomography-Guided versus Angiography-Guided Coronary Stent Implantation trial
IRIS-MAIN	Interventional Research Incorporation Society-Left Main Revascularization registry
IVUS	Intravascular ultrasound
JACC	Journal of the American College of Cardiology
LAD	Left anterior descending artery
LCx	Left circumflex artery
LM	Left main
LMCA	Left main coronary artery
MACE	Major adverse cardiac events
MACCE	Major adverse cardiac and cerebrovascular events
MI	Myocardial infarction
MV	Main vessel
NACE	Net adverse clinical events
NSTE-ACS	Non-ST-elevation acute coronary syndrome
OCT	Optical coherence tomography
OCTOBER	OCT or Angiography Guidance for PCI in Complex Bifurcation Lesions trial
PARIS	Patterns of Non-Adherence to Anti-Platelet Regimens in Stented Patients
PCI	Percutaneous coronary intervention
PRECISE-DAPT	Predicting Bleeding Complications in Patients Undergoing Stent Implantation and Subsequent Dual Antiplatelet Therapy
PRISMA	Preferred Reporting Items for Systematic Reviews and Meta-Analyses
RCT	Randomized controlled trial
RoB 2	Risk of Bias 2
ROBINS-I	Risk Of Bias In Non-randomized Studies-of Interventions
rSS	Residual SYNTAX score
SAPT	Single antiplatelet therapy
SB	Side branch
STEMI	ST-elevation myocardial infarction
SYNTAX	Synergy Between Percutaneous Coronary Intervention With TAXUS and Cardiac Surgery
TIMI	Thrombolysis in Myocardial Infarction
TLF	Target lesion failure
T-PASS	Ticagrelor Monotherapy After Short-Term Dual Antiplatelet Therapy in Acute Coronary Syndrome trial
