# Supplementary material for: Optimal Duration of Dual Antiplatelet Therapy After Percutaneous Coronary Intervention of the Left Main Coronary Artery: A Contemporary Narrative Review
Source: Medicina (Kaunas). 2026 Aug 1;62(8):1487. doi: 10.3390/medicina62081487 (PMC13515272; doi:10.3390/medicina62081487)
Supplement: Supplementary file 1 [file medicina-62-01487-s001.zip › medicina-4451501-supplementary.pdf]

Table S1. Randomized trials informing dual antiplatelet therapy (DAPT) decision-making after left main (LM) percutaneous coronary intervention: LM sample size, antiplatelet comparison, LM-specific analysis, and directness of evidence.

Of the 38 randomized controlled trials included in this review, none was designed primarily to compare DAPT durations exclusively in an LM population. Only IDEAL-LM randomized antiplatelet duration in a 100% LM cohort (confounded with stent platform), and only PRODIGY provided a prespecified subgroup (LM combined with proximal LAD) randomized to differing durations. Trials that enrolled 100% LM patients (EXCEL, NOBLE, DKCRUSH-V, EBC MAIN) randomized revascularization mode or stenting technique rather than DAPT duration, while the large contemporary duration/de-escalation trials (e.g., PARTHENOPE, TWILIGHT, TICO, T-PASS, SMART-CHOICE, STOPDAPT-2/ACS, OPTION, NEO-MINDSET) enrolled broad PCI/ACS populations in which LM patients were a small, generally unquantified subgroup — and MASTER DAPT excluded LM procedures altogether. The evidence base for DAPT duration specifically in LM PCI is therefore predominantly indirect and extrapolated.

Trial (ref.)	Total N / population	LM sample size (n, %)	Randomized comparison (DAPT / other)	LM-specific analysis	Directness of evidence for LM DAPT duration
**A. Trials that randomized antiplatelet duration/strategy within an LM-enriched population — the only DAPT-duration evidence that is even partly LM-direct**					
**IDEAL-LM** (25)	818 patients with LM disease; stable/low-risk	**818 (100%)**	BP-PtCr-EES + 4-mo DAPT vs DP-CoCr-EES + 12-mo DAPT	Dedicated LM randomized cohort; DAPT duration randomized (bundled with stent platform); underpowered for rare events	**Direct**<br>Only trial randomizing DAPT duration in an exclusively LM population; duration confounded with stent type.
**PRODIGY** (40)	1,754 all-comers; prespecified anatomy subgroup	**953 LM or proximal-LAD** (54%)	6-mo vs 24-mo DAPT (subgroup randomized to duration)	Prespecified subgroup by lesion location; 24-mo halved stent thrombosis in LM/pLAD (HR 0.45); P-interaction 0.002	**Partly direct**<br>LM combined with proximal LAD — not LM-isolated; strongest duration signal for the LM/pLAD phenotype.
**B. Trials enrolling 100% LM (or LM-bifurcation) patients that randomized revascularization mode or stenting technique — NOT DAPT duration**					
**EXCEL** (7)	1,905 LM, SYNTAX ≤32	**1,905 (100%)**	PCI (everolimus-EES) vs CABG	LM randomized trial; DAPT held constant per protocol, not compared	**Indirect**<br>Defines PCI eligibility in LM; contributes no DAPT-duration comparison.
**NOBLE** (8, 9)	1,201 LM (88% biolimus-DES); 10-y data	**1,201 (100%)**	PCI vs CABG	LM randomized trial; DAPT not a randomized variable	**Indirect**<br>Final 10-y survival supports PCI in LM; no DAPT-duration arm.
**DKCRUSH-V** (30)	Distal LM bifurcation cohort (N not stated in review)	**100% LM bifurcation**	DK-crush vs provisional stenting (technique, not DAPT)	LM-bifurcation randomized trial; antiplatelet regimen not randomized	**Indirect**<br>Informs stent technique/complexity that modulates DAPT need, not duration itself.
**EBC MAIN** (31)	467 true distal LM bifurcation	**467 (100%)**	Stepwise provisional vs systematic 2-stent (technique)	LM-bifurcation randomized trial; DAPT not randomized	**Indirect**<br>Technique trial; relevant to complexity-based DAPT selection only.
**C. DAPT-duration / de-escalation strategy trials in broad PCI or ACS populations — LM a small, usually unreported subgroup (or explicitly excluded). Evidence applied to LM by extrapolation**					
**PARTHENOPE** (6)	2,107 PCI (mixed CCS/ACS)	Not reported (LM subset included, not quantified)	Score-based personalized DAPT (3/6/24 mo) vs fixed 12 mo	No LM-specific subgroup reported	**Indirect**<br>Principal new duration evidence; personalization framework extrapolated to LM.
**TWILIGHT** (19)	7,119 high-risk PCI	Not reported (complex-PCI subgroup only)	3-mo DAPT → ticagrelor monotherapy vs 15-mo DAPT	Complex-PCI subgroup; no LM-specific analysis	**Indirect**<br>Applied to complex LM by analogy with the complex-PCI subgroup.
**MASTER DAPT** (20)	4,434 high bleeding risk	**LM procedures excluded**	1-mo DAPT → SAPT vs ≥3-mo DAPT	LM PCI excluded by protocol	**Indirect**<br>Explicitly excludes LM; extrapolable only with caution to LM-HBR.
**TICO** (21)	3,056 ACS (ultrathin SES)	Not reported (LM under-represented)	3-mo DAPT → ticagrelor monotherapy vs 12-mo DAPT	No LM-specific subgroup	**Indirect**

Trial (ref.)	Total N / population	LM sample size (n, %)	Randomized comparison (DAPT / other)	LM-specific analysis	Directness of evidence for LM DAPT duration
					ACS de-escalation signal extrapolated to LM.
**T-PASS** (22)	2,850 ACS	Not reported	Ticagrelor monotherapy <1 mo vs ticagrelor+ASA 12 mo	No LM-specific subgroup	**Indirect** Very-early aspirin withdrawal; LM applicability inferred.
**SMART-CHOICE** (46)	2,993 PCI (mixed CCS/ACS)	Not reported	3-mo DAPT → P2Y12 monotherapy vs 12-mo DAPT	No LM-specific subgroup	**Indirect** Monotherapy transition extrapolated to uncomplicated LM.
**STOPDAPT-2 ACS** (45)	4,169 ACS (CoCr-DES)	Not reported	1–2-mo DAPT → clopidogrel monotherapy vs 12-mo DAPT	No LM-specific subgroup	**Indirect** Non-inferiority not met in ACS; cautions against ultra-short DAPT in LM.
**STOPDAPT-2** (48)	PCI, chronic coronary disease	Not reported	1-mo DAPT → clopidogrel vs 12-mo DAPT	No LM-specific subgroup	**Indirect** Short-DAPT reference in stable disease; LM inferred.
**OPTION** (47)	4,551 troponin-negative DES (China)	Not reported	Indobufen + clopidogrel vs ASA + clopidogrel × 12 mo	No LM-specific subgroup	**Indirect** Aspirin-alternative regimen; LM relevance emerging, not tested.
**NEO-MINDSET** (41, 42)	3,410 ACS (post-PCI)	Not reported	Potent P2Y12 monotherapy from discharge vs 12-mo DAPT	STEMI vs NSTE-ACS substudy; no LM-specific subgroup	**Indirect** Non-inferiority not met; cautions against immediate ASA withdrawal in LM.
**D. P2Y12-agent selection and de-escalation trials — inform which agent, not how long; no LM-directed randomization**					
**ISAR-REACT 5** (17)	ACS undergoing PCI (agent comparison)	Not reported	Prasugrel vs ticagrelor (agent, not duration)	No LM-specific subgroup; LM cases embedded in ACS population	**Indirect** Underpins agent preference in ACS incl. LM; not a duration study.
**TROPICAL-ACS** (62)	ACS, guided de-escalation	Not reported	Guided prasugrel→clopidogrel de-escalation vs standard DAPT	No LM-specific subgroup	**Indirect** De-escalation safety extrapolated to stabilized LM-ACS.
**TALOS-AMI** (63)	Stabilized MI (post-hoc)	Not reported	Unguided de-escalation vs continued potent DAPT	Post-hoc analysis; no LM-specific subgroup	**Indirect** Post-hoc de-escalation signal; lowest LM directness.
**E. Intravascular-imaging and bifurcation-technique trials with LM subgroups — optimize the procedure, not DAPT duration**					
**OCTOBER** (13)	1,201 complex bifurcation PCI	**~19% LM bifurcation (≈228)**	OCT-guided vs angiography-guided PCI	Consistent in LM subgroup but not powered for LM-specific conclusions	**Indirect** Imaging optimization; hypothesis-generating for shorter DAPT, not confirmatory.
**ILUMIEN IV** (14)	2,487 diabetes/complex lesions	Not reported (small LM subset)	OCT-guided vs angiography-guided PCI	No dedicated LM subgroup; primary clinical endpoint not met	**Indirect** Larger MSA / less stent thrombosis; no LM DAPT implication tested.
**DEFINITION II** (32)	Complex coronary bifurcation	Not reported (not LM-exclusive)	Systematic 2-stent vs provisional (technique)	No LM-specific subgroup	**Indirect** Complexity/technique context for DAPT selection only.

Directness classification (evidence for DAPT duration in LM PCI).
Direct = DAPT duration randomized within an LM population. Partly direct = DAPT duration randomized in a prespecified subgroup that includes but is not limited to LM. Indirect = LM-specific DAPT duration not randomized (revascularization/technique/imaging trial, agent-selection trial, or broad PCI/ACS trial with LM as an unquantified or excluded subgroup).

“Not reported” indicates the trial did not publish an LM-specific sample size or subgroup. Reference numbers correspond to the main-text reference list. *Abbreviations:* ACS, acute coronary syndrome; ASA, acetylsalicylic acid; BP-PtCr-EES, bioresorbable-polymer platinum-chromium everolimus-eluting stent; CABG, coronary artery bypass grafting; CCS, chronic coronary syndrome; CoCr, cobalt-chromium; DAPT, dual antiplatelet therapy; DES, drug-eluting stent; DP-CoCr-EES, durable-polymer cobalt-chromium everolimus-eluting stent; HBR, high bleeding risk; HR, hazard ratio; LAD, left anterior descending; LM, left main; MSA, minimum stent area; NSTE-ACS, non-ST-elevation ACS; OCT, optical coherence tomography; PCI, percutaneous coronary intervention; SAPT, single antiplatelet therapy; SES, sirolimus-eluting stent; STEMI, ST-elevation myocardial infarction.
